# Near-real-time global gridded daily CO_2_ emissions

**DOI:** 10.1016/j.xinn.2021.100182

**Published:** 2021-11-02

**Authors:** Xinyu Dou, Yilong Wang, Philippe Ciais, Frédéric Chevallier, Steven J. Davis, Monica Crippa, Greet Janssens-Maenhout, Diego Guizzardi, Efisio Solazzo, Feifan Yan, Da Huo, Bo Zheng, Biqing Zhu, Duo Cui, Piyu Ke, Taochun Sun, Hengqi Wang, Qiang Zhang, Pierre Gentine, Zhu Deng, Zhu Liu

**Affiliations:** 1 Department of Earth System Science, Tsinghua University, Beijing 100084, China; 2 Key Laboratory of Land Surface Pattern and Simulation, Institute of Geographical Sciences and Natural Resources Research, Chinese Academy of Sciences, Beijing 100101, China; 3 Laboratoire des Sciences du Climat et de l'Environnement, LSCE/IPSL, CEA-CNRS-UVSQ, Université Paris-Saclay, Gif-sur-Yvette, France; 4 Department of Earth System Science, University of California, Irvine, CA, USA; 5 European Commission, Joint Research Centre (JRC), Ispra, Italy; 6 Key Laboratory of Marine Environment and Ecology, and Frontiers Science Center for Deep Ocean Multispheres and Earth System, Ministry of Education, Ocean University of China, Qingdao 266100, China; 7 Institute of Environment and Ecology, Tsinghua Shenzhen International Graduate School, Tsinghua University, Shenzhen 518055, China; 8 Department of Earth and Environmental Engineering, Columbia University, New York, NY, USA

**Keywords:** near real time, gridded CO_2_ emission, daily, 2020, global change

## Abstract

Precise and high-resolution carbon dioxide (CO_2_) emission data is of great importance in achieving carbon neutrality around the world. Here we present for the first time the near-real-time Global Gridded Daily CO_2_ Emissions Dataset (GRACED) from fossil fuel and cement production with a global spatial resolution of 0.1° by 0.1° and a temporal resolution of 1 day. Gridded fossil emissions are computed for different sectors based on the daily national CO_2_ emissions from near-real-time dataset (Carbon Monitor), the spatial patterns of point source emission dataset Global Energy Infrastructure Emissions Database (GID), Emission Database for Global Atmospheric Research (EDGAR), and spatiotemporal patters of satellite nitrogen dioxide (NO_2_) retrievals. Our study on the global CO_2_ emissions responds to the growing and urgent need for high-quality, fine-grained, near-real-time CO_2_ emissions estimates to support global emissions monitoring across various spatial scales. We show the spatial patterns of emission changes for power, industry, residential consumption, ground transportation, domestic and international aviation, and international shipping sectors from January 1, 2019, to December 31, 2020. This gives thorough insights into the relative contributions from each sector. Furthermore, it provides the most up-to-date and fine-grained overview of where and when fossil CO_2_ emissions have decreased and rebounded in response to emergencies (e.g., coronavirus disease 2019 [COVID-19]) and other disturbances of human activities of any previously published dataset. As the world recovers from the pandemic and decarbonizes its energy systems, regular updates of this dataset will enable policymakers to more closely monitor the effectiveness of climate and energy policies and quickly adapt.

## Public summary

• We present the first near-real-time Global Gridded Daily CO_2_ Emissions Dataset (GRACED)
• GRACED can be updated in near real time with a spatial resolution of 0.1° and a temporal resolution of 1 day
• GRACED shows gridded emissions of seven sectors: power, industry, residential consumption, ground transport, domestic aviation, international aviation, and international shipping
• Regular updates of GRACED will enable policymakers to more closely monitor the effectiveness of climate and energy policies and quickly adapt on various spatial scales

## Graphical abstract

**Figure:**
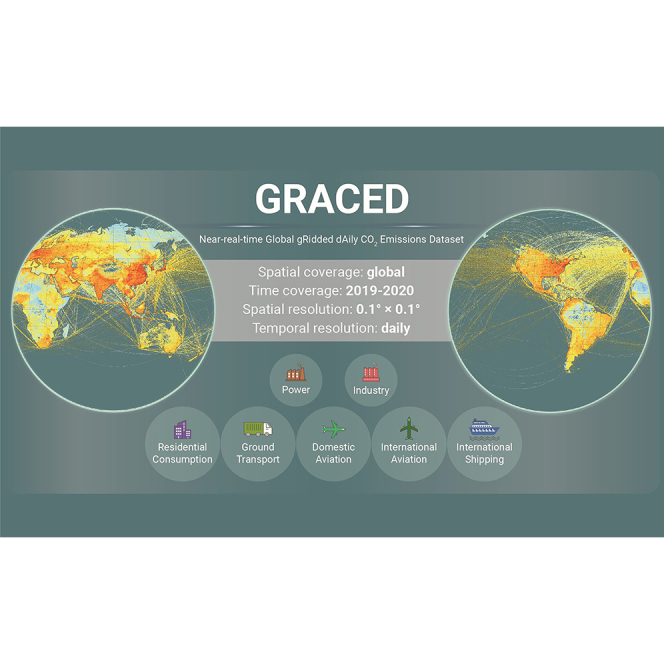

## Introduction

Although human emissions of carbon dioxide (CO_2_) to the atmosphere are the main cause of global climate change, detailed and spatially explicit estimates of such emissions are updated infrequently, typically lagging emissions by at least a year. However, with the rising ambition of climate policies and mitigation efforts,^1^^,^^2^ a reliable, spatially explicit, and up-to-date dataset of fossil CO_2_ emissions is becoming increasingly important. For example, such detailed data are necessary to link emissions to observable atmospheric concentration signals and constrain regional CO_2_ fluxes, and can help decision makers to more quickly assess both the effectiveness of policies and local priorities for further mitigation.^3^^,^^4^

Since the end of 2019, the coronavirus disease 2019 (COVID-19) pandemic has caused major disruptions of human activities and energy use. Governments around the world have imposed compulsory lockdowns that restrict in-person educational and commercial activities to reduce the spread of coronavirus. In turn, industries and factories reduced their activities and production, people's local and long distance mobility was reduced, and human activities were reduced on a large scale, resulting in a substantial decrease in fossil energy consumption and CO_2_ emissions, albeit with large regional differences.^4^, ^5^, ^6^ As lockdown restrictions have relaxed in many countries and economic activities have recovered in some sectors, the effect of the pandemic on CO_2_ emissions has weakened, even during large second waves of cases. A timely and finely gridded emissions dataset enables quantitative analysis of temporal and spatial changes in CO_2_ emissions in each country in response to emergencies (e.g., COVID-19) and other disturbances of human activities, and helps to constrain predictions of future trends.

Existing datasets of global gridded (i.e., spatially explicit) CO_2_ emissions include the Open-source Data Inventory for Anthropogenic CO_2_ (ODIAC), which distributes national emission totals estimated by the Carbon Dioxide Information Analysis Center (CDIAC) in space, using a combination of geospatial proxies such as satellite observations of nighttime lights and geolocations of major power plants (Carbon Monitoring for Action list): ODIAC provides maps of monthly CO_2_ emissions on a 1-km grid for the period 2000 to 2019, as of today, including emissions from power plant, transportation, cement production/industrial facilities, and gas flares over land regions.^7^, ^8^, ^9^ Similarly, the Community Emissions Data System (CEDS) uses data from a number of existing inventories to provide a monthly gridded dataset of all emission species for the Climate Model Inter-comparison Program (CMIP6) over the period 1750 to 2014 at a resolution of up to 0.1°, including sectors of energy transformation and extraction, industry, residential, commercial, transportation, agriculture, solvent production and application, waste, shipping, and other.^10^, ^11^, ^12^, ^13^ Another prominent example is the Emission Database for Global Atmospheric Research (EDGAR). EDGAR estimates emissions based on national CO_2_ emissions reported by the Global Carbon Project (GCP) and emission factors, broken down to Intergovernmental Panel on Climate Change (IPCC)-relevant source-sector levels. EDGAR uses spatial geospatial proxies such as point and line source locations at a 0.1° × 0.1° resolution for the period 1970 to 2019, including sectors of agriculture, power, transport, residential, industry, manufacturing, and a number of others.^14^, ^15^, ^16^ More recently, The Global Carbon Grid (http://gidmodel.org) establishes high-resolution maps of global CO_2_ emissions from fossil fuel combustion and cement production based on a framework that integrates multiple data flows, including point sources, country-level sectoral activities and emissions, and transport emissions and distributions. The Global Carbon Grid v1.0 provides global 0.1° × 0.1° CO_2_ emission maps of six source sectors: power, industry, residential, transport, shipping, and aviation in 2019.^17^, ^18^, ^19^

Even the most current of the gridded CO_2_ emissions datasets described above lag emissions by a year or more and do not reflect sub-monthly temporal variations related to seasonality, weather, economic activities, or policies. Nassar et al. made a first attempt to further downscale these global datasets at the weekly and diurnal scale using static local temporal scaling factors.^20^ However, during a normal year, day-to-day variations are due mainly to weather affecting heating/cooling demands of residences and commercial buildings and the generation of renewable energy, as well as weekends and holidays. Since the pandemic began in early 2020, though, daily variations have been perturbed by a multitude of other factors, including lockdowns, industrial production drops and recoveries, and changes in human behavior. Timely and quantitative analysis on the effects of these COVID-related changes on CO_2_ emissions using tools such as inversion systems thus requires dynamic knowledge of global CO_2_ emissions. It was this need for data that led to our development of the Carbon Monitor, a near-real-time daily dataset of global CO_2_ emission at the national level (https://carbonmonitor.org).^3^^,^^4^ Chevallier et al. disaggregated the daily national Carbon Monitor totals on a worldwide uniform grid using satellite retrievals of a pollutant co-emitted with CO_2_ as a spatial proxy, without sectoral distinction.^21^ Here, we considerably refine the approach by downscaling the daily national emissions from Carbon Monitor into a 0.1° × 0.1° grid for each of the seven sectors (power, industry, residential, ground transportation, domestic aviation, international aviation, and international shipping), using sector-specific geospatial data from the Global Carbon Grid (GID) v1.0, the EDGARv5.0_FT2019 database for 2019, and NO_2_ retrievals from the Tropospheric Monitoring Instrument (TROPOMI) on board the Sentinel-5 Precursor satellite to provide a new spatially explicit dataset of daily global CO_2_ emissions covering the last 2 years since January 1, 2019, which we name GRACED. The first high-resolution near-real-time gridded fossil CO_2_ emission GRACED we presented will facilitate the adaptive management of emissions and the implementation of climate policy, which is of great importance of achieving carbon neutrality around the world.

## Results

### Quarterly mean emissions

**Figure 1:**
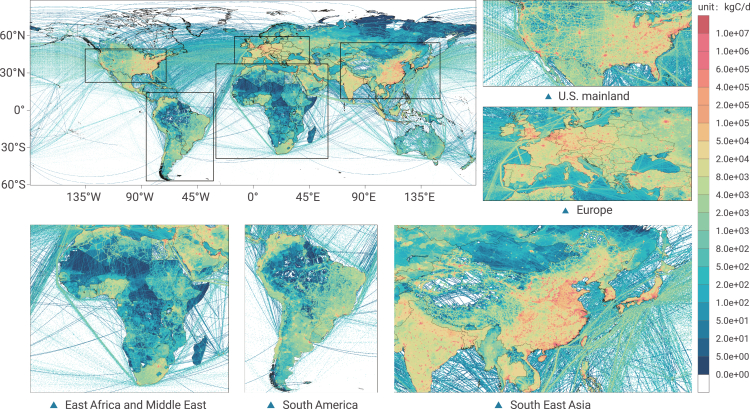
The fossil fuel and cement CO_2_ emissions distributions of GRACED in 2020 The value is given in the unit of kilograms of carbon per day per cell

The global daily average emissions from all sectors of GRACED in 2020 are shown in Figure 1. GRACED demonstrates fine-grained emission differences produced by the allocation of emissions at the sub-national level. Emissions are shown at a common 0.1° × 0.1° resolution. In the figure, the five major global regions (US mainland, Europe, Southeast Asia, East Africa and Middle East, South America) are enlarged and displayed. It is shown that the spatial distribution characteristics of daily average emissions throughout 2020 are clustered, concentrated in areas such as eastern US, western Europe, southeastern China, South Korea, Japan, and India, with megacities as hotspots. The daily average total emissions in 2020 are approximately 3,821 kg of carbon per day (kgC/d) per cell. The cell with the maximum emission value is 41,320 tC per day per cell.

We also calculate quarterly daily average total (Figure S1) and sectoral (Figure S2) emissions of 2020. We define January, February, and March as the first quarter, and then define other months included in other quarters. The average total emission in the first quarter is the highest, with 3,969 kgC per day per cell, and the average total emission quarter is the lowest in the second quarter, with 3,381 kgC per day per cell.

As about 90% of the world's population is located in the northern hemisphere, the level of human activities in the northern hemisphere dominates the values of global emissions. The residential consumption sector and the aviation sectors generate the most emissions in the fourth quarter, resulting in the highest average total emissions in the first quarter. Except for the residential consumption, industrial, and international shipping sectors, the average lowest emissions from the other sectors all appear in the second quarter, which dominates the results of the lowest average total emissions in the second quarter.

### Difference between weekend and weekday emissions

**Figure 2:**
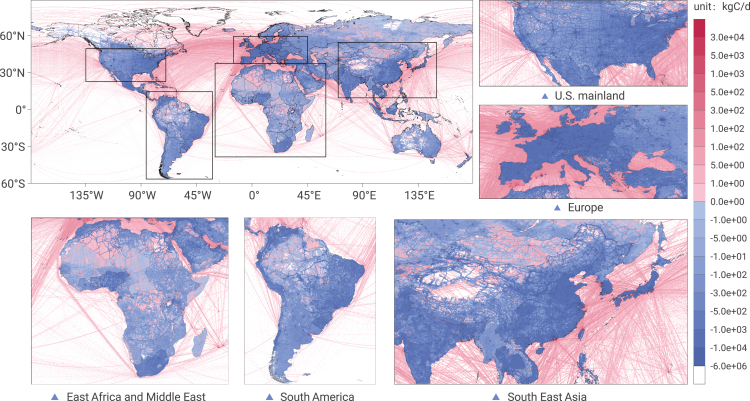
Map of weekend minus weekday emissions in 2020

We then investigate the difference between weekend emissions and weekday emissions in Figure 2. It can be seen that, on average, the global CO_2_ emissions on weekends are generally less than the CO_2_ emissions on weekdays. The global average of this difference is −248 kgC per day per grid. It can be further seen that the more developed regions have more significant differences between weekdays and weekends than the less developed regions (shown as the dark blue areas in the figure). Moreover, the spatial distribution characteristics of this difference showed an obvious linear relationship with the ground transportation sector's emission. It indicates that the reduction of human driving activities on weekends has a very important impact on the reduction of weekend emissions.

In 2019, on average, the global carbon emission on weekends was generally less than the emission on weekdays (Figure S7). The average value of this difference is −303 kgC per day per grid globally, which is higher compared with 2020. This is mainly because, affected by COVID-19 in 2020, general human travel has generally reduced under the lockdown measures; at the same time, the implementation of the home office policy has weakened commuting travel during weekdays, making the difference in emissions between weekends and weekdays in 2020 less significant.

### Emission changes due to COVID-19

**Figure 3:**
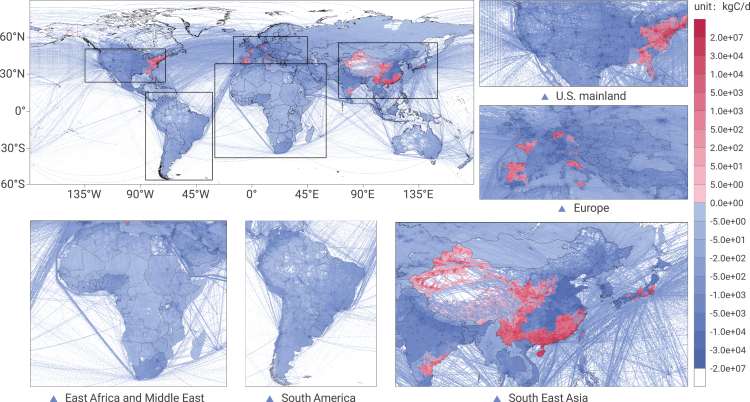
Difference in daily average CO_2_ emissions between 2020 and 2019 (2020 minus 2019)

Affected by the COVID-19 pandemic in 2020, compared with 2019, total emissions have generally declined worldwide (Figure 3). There were, however, a few regions experiencing an emission increase, such as the eastern US, the United Kingdom, some areas of Europe, southeastern India, some of Japan's provinces, and central and western China. For percentage change information, please see Figure S8 for the details.

**Figure 4:**
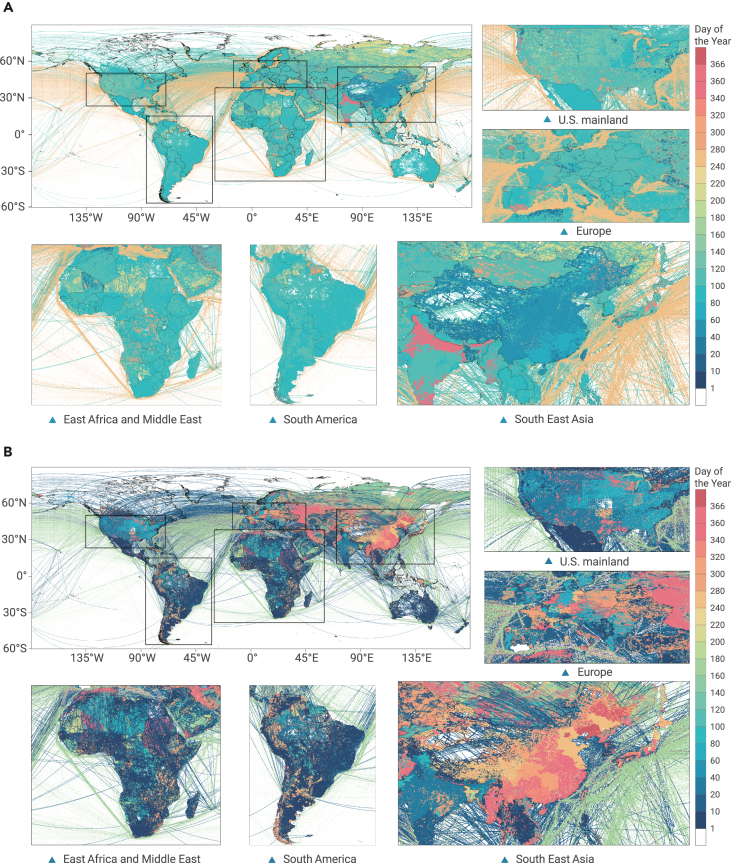
The day of the year with the biggest change in 2020 compared with 2019 (A and B) The day of the year with (A) the maximum reduction, and (B) the maximum rebound of each grid in 2020 compared with 2019.

The dates with the maximum reduction and the maximum rebound in different regions in 2020 compared with 2019 reflect the sequence of the significant reduction in human activities caused by the severe impact of COVID-19 (Figure 4A). In this study, we define the date with the maximum rebound as the date that appears to have the biggest increase in emissions in 2020 compared with 2019. The dates with the maximum rebound in different regions in 2020 compared with 2019 reflect the sequence of the largest economic recovery in the later period (Figure 4B).

In Figure 4A, obviously, some international aviation and international shipping was the first to be hit, which is shown in dark blue lines. From a national perspective, China's largest decline in 2020 appeared earliest compared with other countries, and timewise is closely related to China's first hit by COVID-19, while, the US, Spain, and other countries experienced the largest emission reduction later. Most regions of India and Japan experienced this situation soon after. This may be mainly related to the late impact of the first wave of COVID-19 in these countries and the more severe impact of COVID-19's second wave in the later period.

Judging from the date with the largest rebound (Figure 4B), China; Russia; Myanmar; some European countries, such as the Netherlands, Poland, and Italy; and some other countries experienced the largest rebound later, while, in India; some states in the US, and some European countries, such as Spain, Belarus, and Ukraine, the biggest rebound occurred earlier.

### Sectoral emissions share

**Figure 5:**
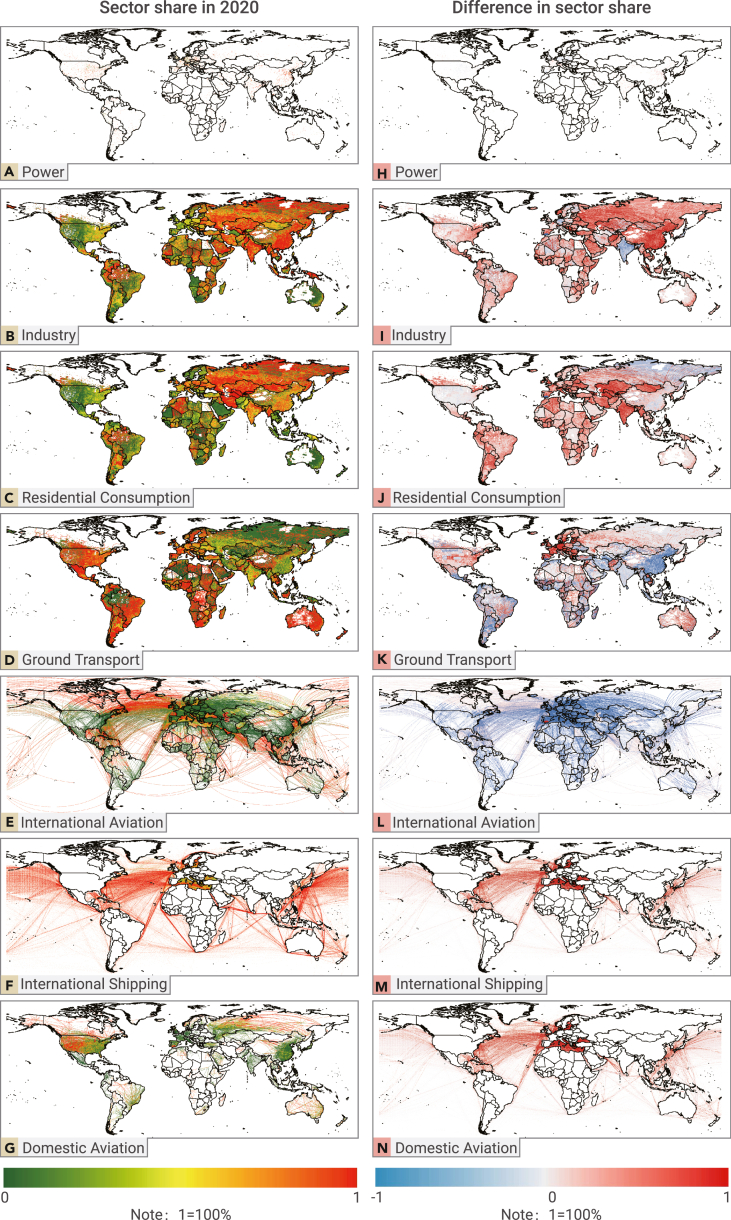
Sector share of CO_2_ emissions and its difference between 2020 and 2019 (A–G) Sector share of CO_2_ emissions in 2020 (A–G). (H–N) Difference in sector share of CO_2_ emissions between 2020 and 2019 (2020 minus 2019) (H–N).

Different sectors exhibit various spatial patterns. The sector share of  CO_2_ emissions in 2020 and its difference between 2020 and 2019 are shown in Figure 5. The emissions shares of various sectors in 2020 are shown in Figures 5A–5G. At the grid level, changes in sector share between 2020 and 2019 are also observed (Figures 5H–5N). Please see the sectoral emissions share part in the supplemental information file for the detailed descriptions.

### Uncertainty analysis

The uncertainties are from Carbon Monitor, GID, and EDGAR dataset.

The uncertainty analysis of Carbon Monitor was presented in our related paper recently published at Nature Communications.^4^ We followed the 2006 IPCC Guidelines for National Greenhouse Gas Inventories to conduct an uncertainty analysis of the data. First, the uncertainties were calculated for each sector. The uncertainty ranges of the power, ground transport, industry, residential, aviation, and international shipping sector are ±14.0%, ±9.3%, ±36.0%, ±40.0%, ±10.2%, and ±13.0%, respectively. The uncertainty in the emission projection for 2019 is estimated as 2.2% by combining the reported uncertainty of the projected growth rates and the EDGAR estimates in 2018. Then, we combine all the uncertainties by following the error propagation equation from the IPCC. Equation 5 shows that the overall uncertainty range of Carbon Monitor is ±7.2%.

As for GID and EDGAR, uncertainty is introduced in the magnitude of national-level total emissions, the magnitude and location of large point sources, the magnitude and distribution of non-point sources, and from the use of proxy data to characterize emissions. As pointed out by Hogue et al., the largest uncertainty contribution in gridded emission datasets comes from how well the distribution of the proxy used for spatial disaggregation represents the distribution of emissions.^22^ So, for the gridded data from GID and EDGAR used in this research, the largest contribution to uncertainty comes from the spatial disaggregation process of national-level emissions and the accuracy of the spatial proxy parameters. The subtraction of the sum of all precise point sources with little uncertainty from the national total of a specific sector leaves a remaining emission composed of smaller sources. Due to lack of information, the remaining emission is usually allocated based on, e.g., a population density proxy. The uncertainties of the point sources and the remaining smaller sources are greatly different, being larger than the uncertainty of the national total of a specific sector. The representative information about the selected characteristic parameters of the point sources is most critical and needs to be evaluated by measurements (such as on-site atmospheric measurement of CO_2_ emission pollutants), but in-depth analysis beyond the scope of this paper would be required.

## Discussion

This research presents for the first time the near-real-time high-resolution gridded fossil CO_2_ emissions from fossil fuel and cement production, which is based on the Carbon Monitor project.^4^ In this work, we developed a near-real-time global gridded emission dataset called GRACED to provide a high-quality, fine-grained dataset since January 1, 2019. This dataset is a daily gridded map with a spatial resolution of 0.1° × 0.1°. One of the advantages of GRACED is that it can support global near-real-time carbon emission monitoring on various fine spatial scales (such as cities) at sub-national level, which can further improve our understanding of the spatiotemporal variability in emissions and human activities. Through the long time series of GRACED, we provide important daily-scale input for the analysis of emission trends during the COVID-19 pandemic, which will help to carry out more local and adaptive management of climate change mitigation in post-COVID era.

We found that carbon emissions are mainly concentrated in the eastern US, western Europe, southeastern China, South Korea, Japan, and India spatially. A sharp decline of CO_2_ emissions in 2020 was identified in the central and eastern US, the United Kingdom, France, and Germany in Europe, and in India, Japan, South Korea, and eastern China. Various sectors show different spatial distribution characteristics, which is mainly explained by the emission sources.

In general, the current statistical data cannot fully grasp the fine-grained dynamics of CO_2_ emissions under the COVID-19 pandemic, and further monitoring, observation, and data collecting are urgently needed. The ability of near-real-time fine-grained monitoring of daily emission trends we demonstrate here helps to take timely local actions in regional, sub-national, or urban areas, and has policy implications for local climate change mitigation and earth system management.

GRACED provides the first global near-real-time gridded carbon emissions data. This globality and timeliness comes at the expense of reduced accuracy due to near-real-time spatial allocation information. Therefore, it is recommended that potential users of GRACED carefully consider these limitations when using this dataset. Inevitably, with the updated version of proxy data, the accuracy of emission spatial allocation in future versions of GRACED can be further improved. With Carbon Monitor national-level data and satellite retrievals data publicly updated in near real time, there are no restrictions on continuing to produce updated future versions of GRACED products within the same model framework.

## Materials and methods

### Datasets used in the study

(1) A near-real-time daily dataset of global sectoral CO_2_ emission from fossil fuel and cement production at national level since January 1, 2019, published as Carbon Monitor (data available at https://carbonmonitor.org/).^4^ (2) Global sectoral CO_2_ emissions annual data with high resolution of 0.1° in 2019 based on a framework that integrates multiple data flows, including point sources, country-level sectoral activities and emissions, and transport emissions and distributions released by the Global Carbon Grid (http://gidmodel.org).^17^, ^18^, ^19^^,^^23^, ^24^, ^25^ (3) Global monthly gridded emissions at a 0.1° × 0.1° resolution in 2019 defined for a large number of IPCC sub-sectors provided by the EDGAR (https://edgar.jrc.ec.europa.eu/overview.php?v=50_GHG).^16^^,^^26^ (4) Daily NO_2_ Thermal Chemical Vapor Deposition (TCVD) retrievals data in 2019 and 2020 from the Tropospheric Monitoring Instrument (TROPOMI) on board the Sentinel-5 Precursor satellite, launched in October 2017.

The ground resolution of the TROPOMI NO_2_ retrievals was 7 × 3.5 km^2^ at nadir until 5 August 2019 and has been 5.5 × 3.5 km^2^ since then, achieving near-global coverage in 1 day. Standard retrievals from the official offline processing with a quality assurance value greater than 0.75 were aggregated to daily time scale on a regular 0.1° × 0.1° global grid and averaged over 14-day averaging periods in order to reduce the retrieval noise and limit gaps in the retrievals.

### Spatial gridding methodology

#### Grouping the GID and EDGAR sectors into Carbon Monitor categories

First, we link the Carbon Monitor emission sectors to GID and EDGAR sectors according to Table S1. We consider that GID has the highest accuracy in source location and we rely on this database as much as possible. However, for the domestic aviation, international aviation, and international shipping sectors, GID does not distinguish between related domestic and international sub-sectors: we therefore directly use EDGAR's monthly spatial patterns for the spatial distribution in these sectors.

#### Spatially gridding procedure

Second, we do a spatially gridding procedure. We use the global annual spatial patterns of CO_2_ emission from the GID sub-sectors and global monthly CO_2_ emission spatial patterns from EDGAR sub-sectors for the year 2019 for spatially downscaling Carbon Monitor daily national-level emissions. We assume that the spatial pattern of emissions remained unchanged after the last year of GID and EDGAR (2019). The validity of this assumption will depend on the country and on the time horizon for the adjustment, while the sub-national emission may change rapidly within a country from 2019 to 2020 as there was a great difference in the timing and degree of the impact of COVID-19 in various regions. Therefore, for large emitters that have a significant impact on global total emissions, we use sub-national proxy based on TROPOMI NO_2_ retrievals data to allocate national carbon emission totals into regional totals, before doing a second downscaling at 0.1° based on the GID and EDGAR spatial patterns. The analysis can be updated consistently with the latest high-resolution emission maps and other spatial proxies for each year.

**Figure 6:**
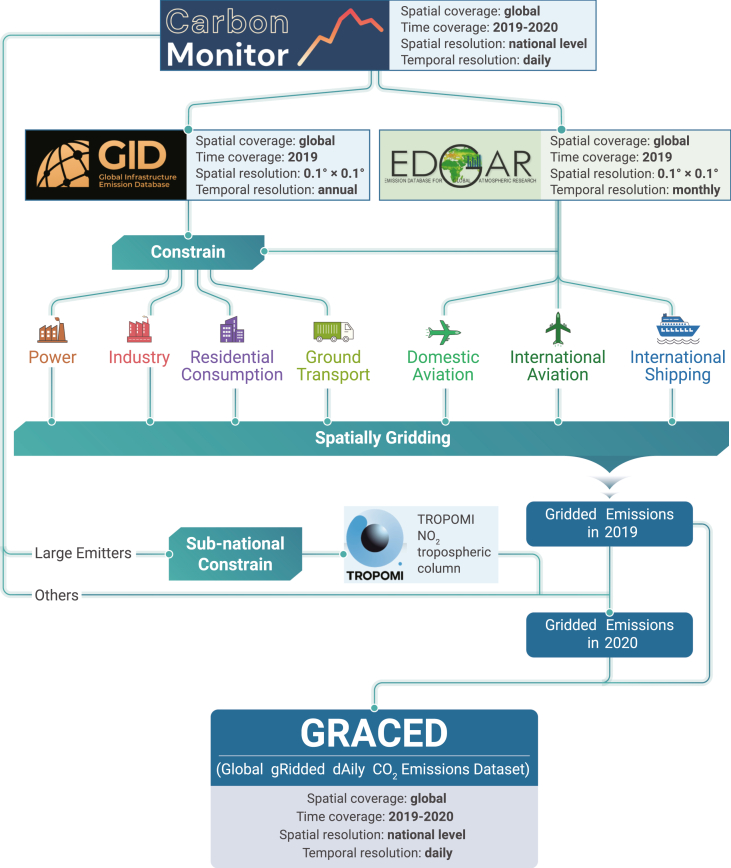
The framework of top-down spatially gridding methodology

The spatial disaggregation framework used in the GRACED is shown in Figure 6. It is a top-down methodology that allocates Carbon Monitor national-level daily emissions to finer-grid cells using spatial patterns provided by GID and EDGAR and sub-national proxy based on TROPOMI NO_2_ retrievals.

The detailed process of the model is presented as follows:

(1) First, we use the spatial patterns provided by GID to allocate the national-level emissions of the four sectors of Carbon Monitor (power, industry, residential consumption, ground transport sector; see Table S1) from Carbon Monitor to obtain the daily gridded emissions under GID's annual spatial patterns. We then integrate the monthly spatial patterns of EDGAR for further correction, to correct the previous daily gridded emission based on GID's annual spatial patterns under the monthly spatial patterns. For the domestic aviation sector, international aviation sector, and international shipping sector, as GID does not distinguish between related domestic and international sub-sectors compared with EDGAR, we directly use EDGAR's monthly spatial patterns for distribution. The first version value of emission $Emi\_v1_{g,d,s}$ for grid *g*, date *d*, and sector *s* is:

(Equation 1) $$Emi\_v1_{g,d,s1}=CM_{country,d,s1}*\frac{GID_{g,s1}}{\sum_{i=1}^{n}GID_{i,s1}}*\frac{EDGAR_{g,m,s1}}{\sum_{j=1}^{12}EDGAR_{g,j,s1}}*12$$

(Equation 2) $$\begin{array}{l} Emi\_v1_{g,d,s2}=CM_{country,d,s2}*\frac{EDGAR_{g,m,s2}}{\sum_{i=1}^{n}EDGAR_{i,m,s2}} \end{array}$$

where $CM_{country,d,s}$ means the value of Carbon Monitor for country *country* that grid point *g* belongs to, day *d*, and sector *s*. *s1* belongs to one of the sectors power, industry, residential consumption, and ground transport. *s2* includes international aviation, domestic aviation, and international shipping. $GID_{g,s1}$ means the value of GID gridded CO_2_ emission for grid point *g* and sector *s1*. *n* is the total number of grid points within this country. $EDGAR_{g,m,s}$ means the value of EDGAR for grid point *g*, month *m* that date *d* belongs to, and sector *s*. *j* is the index of a month.
(2) For large emitters, sub-national emission patterns can vary significantly from one year to the next, which has a great impact on the global total emissions. This was particularly obvious in 2020 with regional variations in the COVID-19 crisis, for instance, between eastern and western US, or between eastern and western China. Capturing those sub-national emission changes is important to having a competitive product that could avoid the negative impact on the dataset's accuracy caused by ignoring significant variations of large emitters' sub-national emission patterns, and is not addressed by Equations 1 and 2, which use a climatological emission pattern. It is reported that the global changes in emissions are also consistent with global changes in the NO_2_ inventory from satellite data.^6^ Therefore, we assume that the sub-national emission changes follow the pattern of the differences in NO_2_ column concentration between 2020 and 2019. In detail, we calculate an index R of each province of large emitters, which is the averaged NO_2_ concentration of each province, according to TROPOMI NO_2_ retrievals data in year *y*:

(Equation 3) $$\begin{array}{l} R_{p,y}=NO_{2_{p,\;y}} \end{array}$$

where *p* represents province (state), and *y* represents the year. $NO_{2_{p,\;y}}$ is the satellite NO_2_ concentration averaged temporally over rolling 14-day period in year *y* for province *p* (as explained above) and spatially over the 5% grid points within each province (state) that has the largest NO_2_ average over the year. The choice of the 5% largest values allows extracting clear patterns very close to emission location. In the following step, we remove any negative NO_2_ value for the 5% grid points over the year 2019 and 2020 that may be generated and attribute the mass gain to the other 5% pixels. Last, we calculate index R of each province in 2019 (2020) according to TROPOMI NO_2_ retrievals data.

Then we generate $CM_{p,d,s1,2020}$, the daily provincial emission in day *d* and for sector *s1* adjusted by the TROPOMI NO_2_ retrievals in day *d* and for sector *s* in 2020 that matches the daily national total from Carbon Monitor following Equation 4:

(Equation 4) $$\begin{array}{l} CM_{p,d,s1,2020}=\frac{CM_{p,d,s1,2019}*R_{p,2020}/R_{p,2019}}{\sum_{p=1}^{np}CM_{p,d,s1,2019}*R_{p,2020}/R_{p,2019}}\times CM_{country,d,s1,2020} \end{array}$$

where $CM_{p,d,s1,2019}$ means the first version of the emission value of a province in day *d* and for sector *s1* in 2019. *np* is the number of provinces of the country. In detail, first, we calculate the ratio of change in the R index in 2020 compared with 2019, which is $R_{p,2020}/R_{p,2019}$. Second, multiply the provincial emission value aggregated from our first version dataset for 2019, $CM_{p,d,s,2019}$, to update the provincial emission value for 2020. Last, divide the updated provincial emission value by the sum of the updated provincial emission value $\sum_{p=1}^{np}CM_{p,d,s1,2019}*R_{p,2020}/R_{p,2019}$ in 2020 to do the normalization processing in the Equation 4. So, the sum of the updated provincial emissions within a country can be consistent with the national-level emission value from Carbon Monitor in 2020 after multiplying the national-level emission $CM_{country,d,s1,2020}$ from Carbon Monitor.

Then, based on the updated provincial emission $CM_{p,d,s1,2020}$ in 2020, we use GID and EDGAR data as the spatial patterns to distribute the emission data of each province for large emitters to obtain our final version gridded emission value $Emi\_v2_{g,d,s}$:

(Equation 5) $$\begin{array}{l} Emi\_v2_{g,d,s_{1}}=CM_{p,d,s1,2020}*\frac{GID_{g,s1}}{\sum_{i=1}^{n}GID_{i,s1}}*\frac{EDGAR_{g,m,s1}}{\sum_{j=1}^{12}EDGAR_{g,j,s1}}*12 \end{array}$$

where *n* means the total number of grids within this province.

After revising the gridded emissions for large emitters Brazil, China, France, Germany, India, Italy, Japan, Spain, US, and UK in 2020, GRACED is finally generated.

## Website

https://scholar.harvard.edu/zhu.

## Supplemental information

Document S1. Supplemental materials and methods, Figures S1–S10, and Tables S1 and S2

Document S2. Article plus supplemental information

## Footnotes

Supplemental information can be found online at https://doi.org/10.1016/j.xinn.2021.100182.

## References

[1] Chen J.M. (2021). Carbon neutrality: toward a sustainable future. The Innovation.

[2] Wang F., Harindintwali J., Yuan Z. (2021). Technologies and perspectives for achieving carbon neutrality. The Innovation.

[3] Liu Z., Ciais P., Deng Z. (2020). Carbon monitor, a near-real-time daily dataset of global CO_2_ emission from fossil fuel and cement production. Nat. Scientific Data.

[4] Liu Z., Ciais P., Deng Z. (2020). Near-real-time monitoring of global CO_2_ emissions reveals the effects of the COVID-19 pandemic. Nat. Commun..

[5] Forster P.M., Forster H.I., Evans M.J. (2020). Current and future global climate impacts resulting from COVID-19. Nat. Clim. Change.

[6] Le Quéré C., Jackson R.B., Jones M.W. (2020). Temporary reduction in daily global CO_2_ emissions during the COVID-19 forced confinement. Nat. Clim. Change.

[7] Oda T., Maksyutov S., Andres R.J. (2018). The open-source data inventory for anthropogenic CO_2_, version 2016 (ODIAC2016): a global monthly fossil fuel CO_2_ gridded emissions data product for tracer transport simulations and surface flux inversions. Earth Syst. Sci. Data.

[8] Oda T., Maksyutov S. (2011). A very high-resolution (1 km×1 km) global fossil fuel CO_2_ emission inventory derived using a point source database and satellite observations of nighttime lights. Atmos. Chem. Phys..

[9] Center for Global Environmental Research, ODIAC (2021). ODIAC Fossil Fuel Emission Dataset.

[10] Hoesly R.M., Smith S.J., Feng L. (2018). Historical (1750–2014) anthropogenic emissions of reactive gases and aerosols from the community emissions data system (CEDS). Geoscientific Model Development.

[11] McDuffie E.E., Smith S.J., O'Rourke P. (2020). A global anthropogenic emission inventory of atmospheric pollutants from sector-and fuel-specific sources (1970–2017): an application of the community emissions data system (CEDS). Earth Syst. Sci. Data.

[12] O'Rourke P.R., Smith S. (2019).

[13] Smith S.J., Zhou Y., Kyle P. (2015). Proceedings of the 2015 International Emission Inventory Conference, San Diego, CA, USA.

[14] Crippa M., Guizzardi D., Muntean M. (2020).

[15] Janssens-Maenhout G., Crippa M., Guizzardi D. (2015). HTAP_v2. 2: a mosaic of regional and global emission grid maps for 2008 and 2010 to study hemispheric transport of air pollution. Atmos. Chem. Phys..

[16] Janssens-Maenhout G., Crippa M., Guizzardi D. (2019). EDGAR v4. 3.2 global atlas of the three major greenhouse gas emissions for the period 1970–2012. Earth Syst. Sci. Data.

[17] Tong D., Zhang Q., Davis S.J. (2018). Targeted emission reductions from global super-polluting power plant units. Nat. Sustainability.

[18] Wang X., Lei Y., Yan L. (2019). A unit-based emission inventory of SO2, NOx and PM for the Chinese iron and steel industry from 2010 to 2015. Sci. Total Environ..

[19] Liu J., Tong D., Zheng Y. (2021). Carbon and air pollutant emissions from China's cement industry 1990–2015: trends, evolution of technologies, and drivers. Atmos. Chem. Phys..

[20] Nassar R., Napier-Linton L., Gurney K.R. (2013). Improving the temporal and spatial distribution of CO_2_ emissions from global fossil fuel emission data sets. J. Geophys. Res. Atmospheres.

[21] Chevallier F., Zheng B., Broquet G. (2020). Local anomalies in the column-averaged dry air mole fractions of carbon dioxide across the globe during the first months of the coronavirus recession. Geophys. Res. Lett..

[22] Hogue S., Marland E., Andres R.J. (2016). Uncertainty in gridded CO_2_ emissions estimates. Earth’s Future.

[23] Meijer J.R., Huijbregts M.A., Schotten K.C., Schipper A.M. (2018). Global patterns of current and future road infrastructure. Environ. Res. Lett..

[24] Zheng B., Huo H., Zhang Q. (2014). High-resolution mapping of vehicle emissions in China in 2008. Atmos. Chem. Phys..

[25] Liu H., Fu M., Jin X. (2016). Health and climate impacts of ocean-going vessels in East Asia. Nat. Clim. Change.

[26] Crippa M., Solazzo E., Huang G. (2020). High resolution temporal profiles in the Emissions Database for Global Atmospheric Research (EDGAR). Nat. Scientific Data.

