# Supplementary material for: Near-real-time global gridded daily CO2 emissions
Source: Innovation (Camb). 2021 Nov 2;3(1):100182. doi: 10.1016/j.xinn.2021.100182 (PMC8703084; doi:10.1016/j.xinn.2021.100182)
Supplement: Document S1. Supplemental materials and methods, Figures S1–S10, and Tables S1 and S2 [file mmc1.pdf]

The Innovation, Volume *3*

Supplemental Information

Near-real-time global gridded daily CO_2 emissions

Xinyu Dou, Yilong Wang, Philippe Ciais, Frédéric Chevallier, Steven J. Davis, Monica Crippa, Greet Janssens-Maenhout, Diego Guizzardi, Efisio Solazzo, Feifan Yan, Da Huo, Bo Zheng, Biqing Zhu, Duo Cui, Piyu Ke, Taochun Sun, Hengqi Wang, Qiang Zhang, Pierre Gentine, Zhu Deng, and Zhu Liu

Supplemental Information

Supplemental Text

Carbon Monitor national-level emissions data

GRACED emissions estimates are based on a near-real-time daily dataset of global CO_2 emission from fossil fuel and cement production since January 1, 2019 published as Carbon Monitor (data available at https://carbonmonitor.org/).[1] Emissions estimates from Carbon Monitor are calculated on a national basis and by sector, gaining from past experiences in constructing annual inventories and newly compiled activity data.[2]

Carbon Monitor calculates daily national CO_2 emissions in five sectors (power, industrial production, ground transport, residential consumption and domestic aviation) and daily international aviation and shipping emissions since January 1, 2019. These numbers are provided for the following countries, groups of countries or regions: China, India, the US, the United Kingdom (UK), France, Germany, Italy, the rest of the European Union, Russia, Japan, Brazil, and the rest of the world. These daily emissions estimates are dynamically and regularly updated with an unprecedented latency of about one month only.

The variety of near real-time activity data used upstream by Carbon Monitor includes hourly electricity generation data from 31 countries, traffic congestion data in 416 cities worldwide, daily maritime and aircraft transportation activity data, monthly production data for cement, steel and other energy intensive industrial products in 62 countries/regions, and previous-year fuel use data corrected for air temperature daily variations for residential and commercial buildings emissions. Altogether, the input activity data for Carbon Monitor directly inform about more than 70% of global power and industry emissions, 85% of ground transportation emissions, and 100% of residential and international bunker emissions, respectively. Carbon Monitor also provides the emissions as an aggregate for the rest of world where data are not directly available but as a way to cover all global CO_2 emissions. Based on these high temporal resolution data, national and global daily carbon dioxide emissions with detailed information in 6 sectors and main countries were finally calculated.

Spatially gridded proxy data

GID v1.0 data. The Global Carbon Grid (http://gidmodel.org) establishes high-resolution maps of global CO_2 emissions from fossil fuel combustion and cement production based on a framework that integrates multiple data flows including point sources, country-level sectoral activities and emissions, and transport emissions and distributions. The Global Carbon Grid v1.0 provides global 0.1°×0.1° CO_2 emission maps of six source sectors, including power, industry, residential, transport, shipping, and aviation. More than half of the global CO_2 emissions in 2019 are estimated as point sources with accurate geographic coordinates.[3-5] Another 16% of global CO_2 emissions are from road transport, which is distributed onto road

atla using the method developed by Zheng et al.[6] The global shipping emissions are estimated using the instantaneous engine power of ships based on a combination of the data from the Automatic Identification System (AIS) and the single-vessel technical specification. The global aviation emissions are estimated using the fuel consumption of global aviation and spatially allocated based on the aviation emission maps from EDGAR.[7] Overall, about 70% of the global CO_2 emissions in the Global Carbon Grid v1.0 are location-based estimates, which lay the foundation for high-resolution emission maps.

EDGAR v5.0_FT2019 data. In GRACED, the spatial allocation of emissions is based on EDGARv5.0_FT2019 gridded activity data underlying CO_2 emissions defined for a large number of IPCC sub-sectors and new geospatial proxies.[7,8]

EDGAR is developed and maintained by the Joint Research Centre of the European Commission and is widely used as default for emission estimates in inventories. EDGAR supports the monitoring of the climate policy implementation, and in particular the Paris Agreement and significantly contributes to the quantification of national inventory guidelines of the Intergovernmental Panel on Climate Change (IPCC) and to the assessment of the GHG budgets at different scales (http://verify.lsce.ipsl.fr/).[8,9] EDGAR emissions are split into sub-sectors specified by the Intergovernmental Panel on Climate Change (IPCC) methodology and spatial proxy data / geospatial data such as point and line source location at a 0.1°×0.1° resolution.[7,8] Driven by the development of scientific knowledge on emission generating processes and by the availability of more recent information, the newest EDGARv5.0_FT2019 dataset includes new spatial proxies to distribute population-related emissions based on the Global Human Settlements Layer product.[9]

EDGAR v5.0_FT2019 includes all fossil CO_2 sources, such as fossil fuel combustion, non-metallic mineral processes (e.g. cement production), metal (ferrous and non-ferrous) production processes, urea production, agricultural liming and solvents use.[9]

We use version EDGARv5.0_FT2019 of the dataset, that includes new spatial proxies to distribute population-related emissions based on the Global Human Settlements Layer product. [9] EDGAR v5.0_FT2019 includes all fossil CO_2 sources, such as fossil fuel combustion, non-metallic mineral processes (e.g. cement production), metal (ferrous and non-ferrous) production processes, urea production, agricultural liming and solvents use. Data are presented for all countries, plus bunker fuels, with monthly emissions provided per main source category, and spatially allocated on a 0.1°×0.1° grid over the globe from 1970 till 2018 (https://edgar.jrc.ec.europa.eu/overview.php?v=50_GHG). [7,8]

TROPOMI NO2 retrievals data. GID and EDGAR only use static subnational patterns and currently do not extend after 2019. By definition, the static subnational patterns cannot represent changes in the spatial distribution of the emissions, for instance linked to regional weather anomalies. COVID-19 exacerbates this limitation for the year 2020. We therefore use changes in the distribution of a short-lived pollutant (NO_2) as observed from satellite to reflect the changes in the human activities that cause CO_2 emissions.[10]

We use the NO_2 tropospheric vertical column density retrieved from TROPOMI. The ground resolution of the TROPOMI NO_2 retrievals was 7 × 3.5 km^2 at nadir until 5 August 2019 and has been 5.5 × 3.5 km^2 since then. Most of the cloud-free locations of the globe are observed each day. As in our previous research, we use the standard retrievals from the official offline processing with a quality assurance value greater than 0.75 in the form of daily 0.1° × 0.1° aggregates and average them over rolling 14-day periods in order to dampen the retrieval noise and reduce gaps in the maps.[11]

Range of daily emission variations

The emission variations in different regions of the world in 2020 are shown in **Figure S3**. In 2020, the global grid average variation value is 4417 kgC/d. From a regional perspective, Europe, U.S., China, Southeast Asian countries, India, Japan, South Korea, etc. all have areas with large emission variation values (shown as red areas), and these areas are mainly distributed in economically developed areas, such as the Beijing-Tianjin-Hebei circle in China, the Yangtze River Delta, the Pearl River Delta of China, and California, Utah, and the eastern coastal areas of the U.S.. The emissions in Africa and South America have smaller variation value in 2020 (shown as blue areas). In 2019, the global average variation value of grids is 2930 kgC/d, which is smaller than that in 2020(**Figure S4**).

A low standard deviation of all daily values in a quarter indicates that the emission values tend to be close to the mean (also called the expected value) of the set, while a high standard deviation indicates that the emission values are spread out over a wider variation.

From a quarterly point of view, on average, the distribution of global emission values in the first quarter of 2020 is the most heterogenous, with an average standard deviation value of 811 kgC per day per cell (**Figure S5**). The distribution of global emission values is the most homogenous in the third quarter, with an average standard deviation value of 625 kgC per day per cell. Besides, there are a standard deviation value of 791 kgC per day per cell in the fourth quarter and 634 kgC per day per cell in the second quarter. We also select a few larger cities, including Los Angeles in the U.S., Paris in Europe, Beijing, Shanghai and Wuhan in Asia, and Johannesburg in Africa, to visualize seasonal changes. Please see **Figure S6** for the details about the differences and similarities in the seasonal changes at the city level.

Sectoral emissions share

Different sectors exhibit various spatial patterns. The emissions shares of various sectors in 2020 are shown in **Figure 6A-G**.

As shown in **Figure 6A**, the emissions share from power generation is generally high in the total emissions of the grid to which it belongs. Particularly, the power emissions share in the Democratic Republic of Congo in Africa is not high. This is mainly because this country is

rich in hydropower resources, with hydropower accounting for almost 90%. For the industry sector, its emission share in developed countries such as the U.S., Australia, and Europe is generally low across the country (shown as the light areas in **Figure 6B**), while in China, India, Russia, Southeast Asia, and Africa, the emission share is relatively high, which is represented by the dark areas in **Figure 6B**. It reveals that the industrial activities of these countries still occupy an important position in their national economic activities in 2020. The development pattern of the residential consumption sector is quite different worldwide, and even within the same country, the emissions share of residential consumption sector varies significantly. This is mainly caused by the difference in regional population distribution and activity levels (**Figure 6C**). As shown in **Figure 6D**, ground transportation emissions account for a relatively high proportion of the total emissions worldwide. While in China, India, and Russia, the proportion to the total emission is not as high. This is mainly because their industry emissions are relatively high, making the share of ground transport emissions relatively low. As shown in **Figure 6E**, the emissions share of the international aviation sector is low in most of the land area, except in northern Africa, where the total emissions are low due to economic underdevelopment, making aviation routes through these regions accounted for a relatively high proportion. As international shipping emissions are only distributed in the marine area and only overlap with the spatial distribution of the international aviation sector, its emissions share is generally high (shown as dark areas in **Figure 6F**). For the domestic aviation sector in **Figure 6G,** its emission share varied significantly over the world. Its share is low in southeastern China, western Europe, and some states in the United States, while it is high in the western and central United States, Canada, Russia, and Australia.

At the grid level, changes in sector share between 2020 and 2019 are also observed (**Figure 6H-N**). The large-scale light areas in **Figure 6H** show that, compared with 2019, emissions share of the power sector in most regions throughout 2020 have declined, while this share in Europe and parts of China have increased. In **Figure 6I**, changes in emissions share from industry showed a decline in Europe and India, but shown an increase in most other regions of the world. At the same time, the changes in CO_2 emissions share from the residential consumption sector is more uniform in **Figure 6J**, with almost all regions increasing from 2019 to 2020, which is not only due to changes in population distribution and changes in residential emissions, but also the reduction in the share of other sectors impacted by the COVID-19 pandemics. The areas where ground transport emissions share increases are concentrated in western Europe, Russia and Middle East, while the decline in the share mainly occurs in southeastern China, U.S., and most of other regions (**Figure 6K**). The changes in emissions share of international aviation sector are quite uniform, with almost all regions decreasing from 2019 to 2020 (**Figure 6L**). In contrast, the international shipping sector shows a developing pattern. Compared with 2019, international shipping emissions share in 2020 in almost all regions showed an increase (**Figure 6M**). For the domestic aviation sector, its emissions share in 2020 in almost all regions showed an increase compared with 2019(**Figure 6N**).

Supplemental Figures

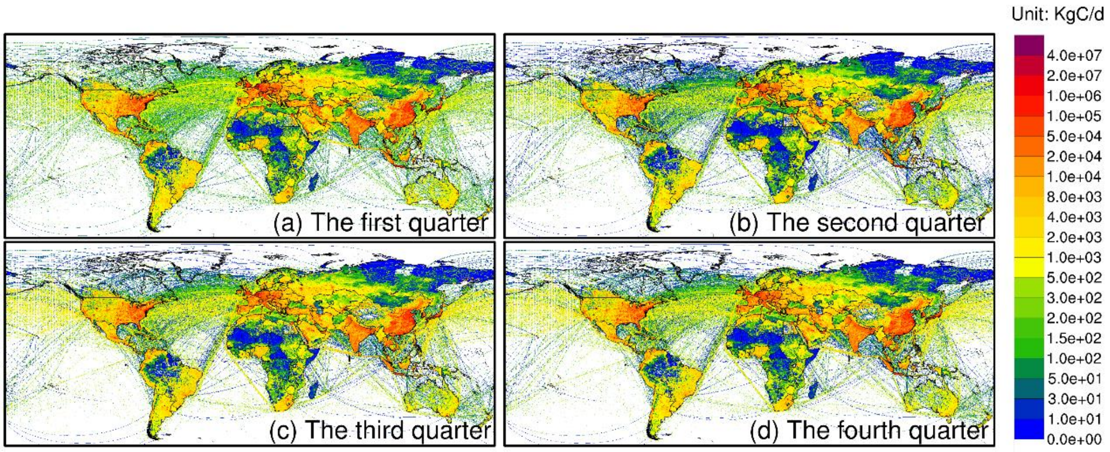

Figure S1. Per quarter daily mean total emissions in 2020.

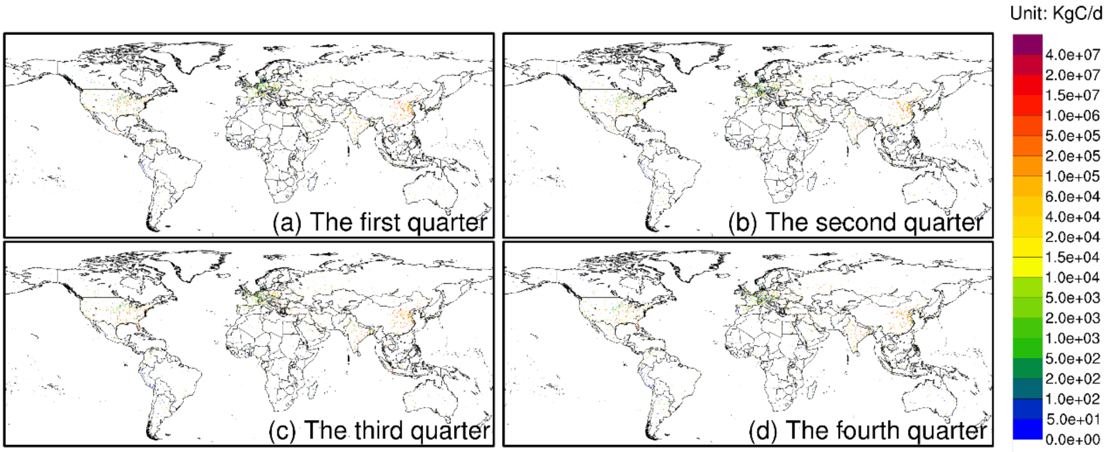

Figure S2(A). Per quarter daily mean emissions from **Power** sector in 2020.

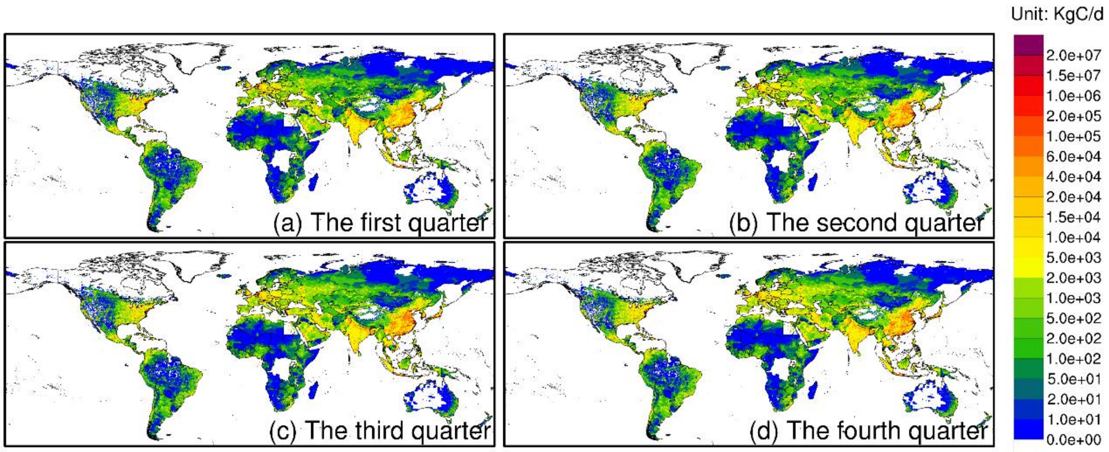

Figure S2(B). Per quarter daily mean emissions from **Industry** sector in 2020.

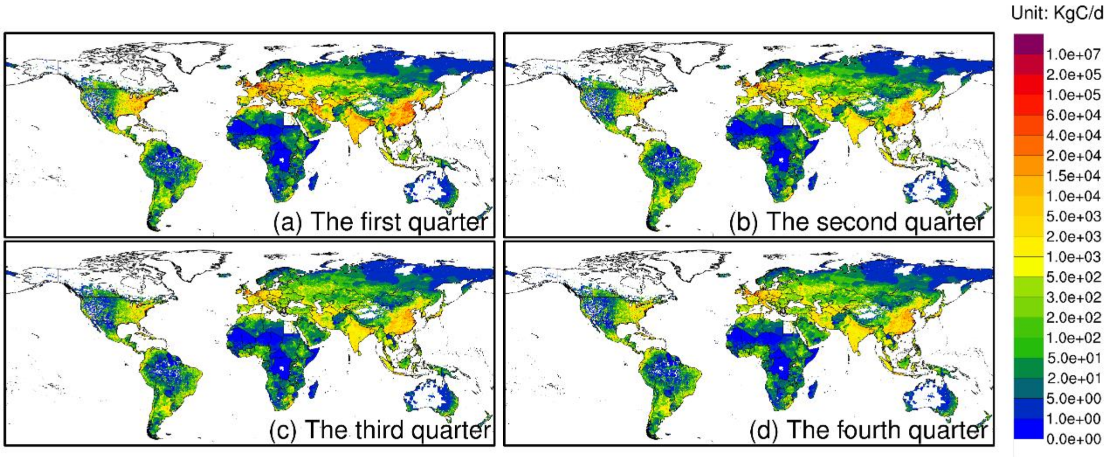

Figure S2(C). Per quarter daily mean emissions from **Residential** sector in 2020.

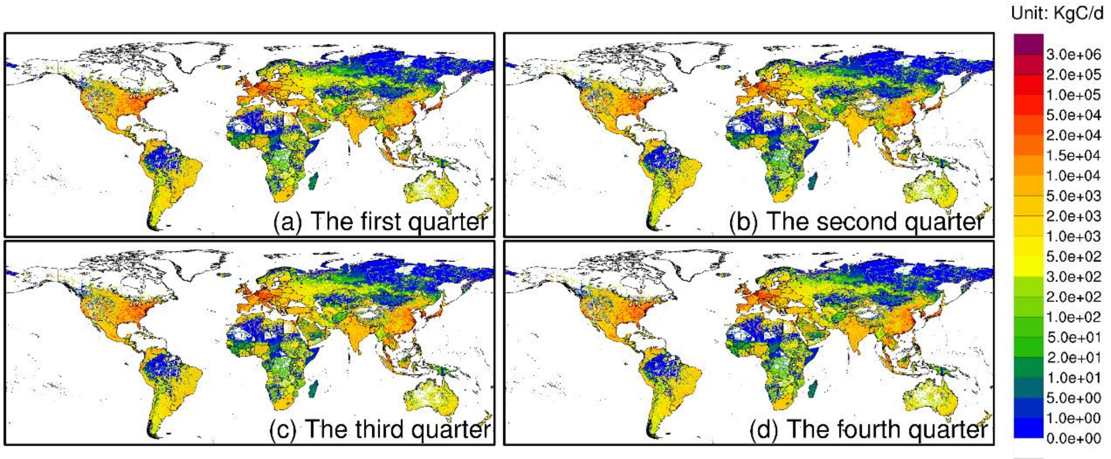

Figure S2(D). Per quarter daily mean emissions from **Ground transport** sector in 2020.

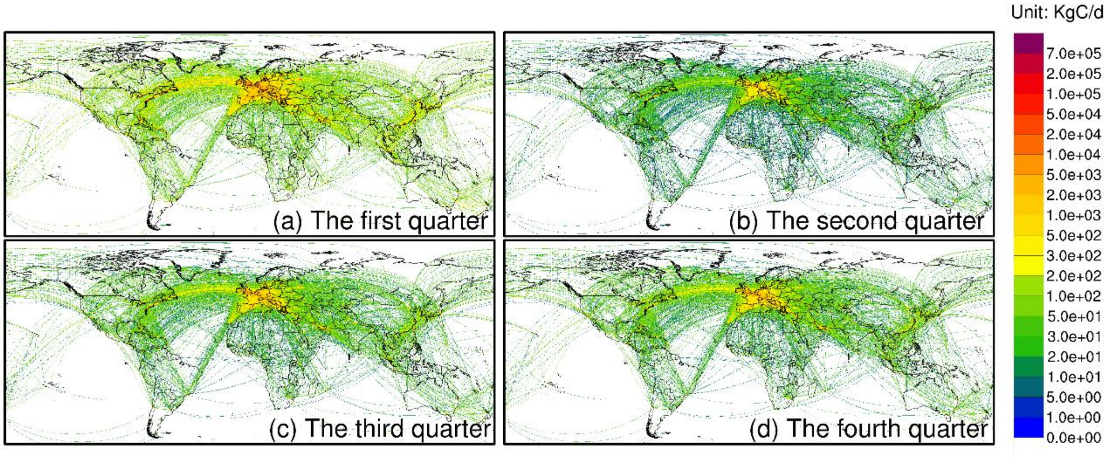

Figure S2(E). Per quarter daily mean emissions from **International aviation** sector in 2020.

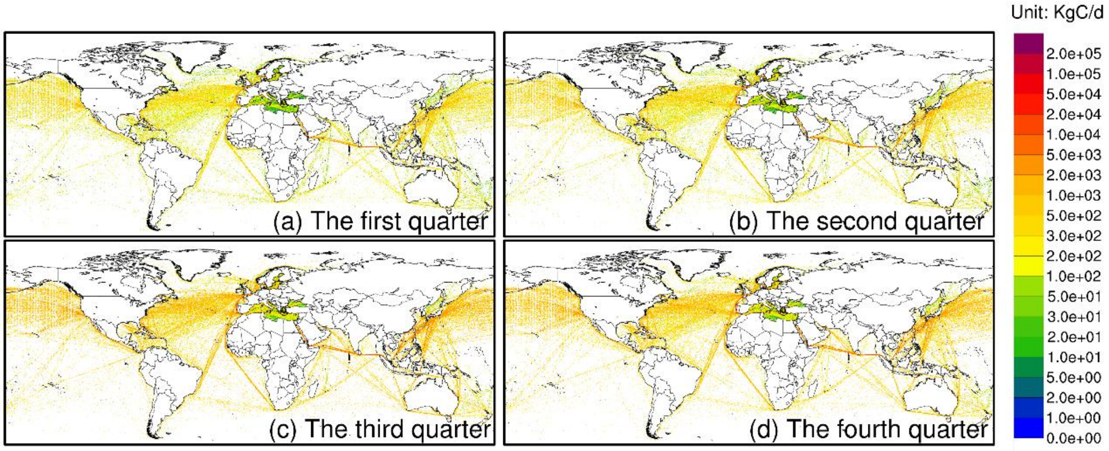

Figure S2(F). Per quarter daily mean emissions from **International shipping** sector in 2020.

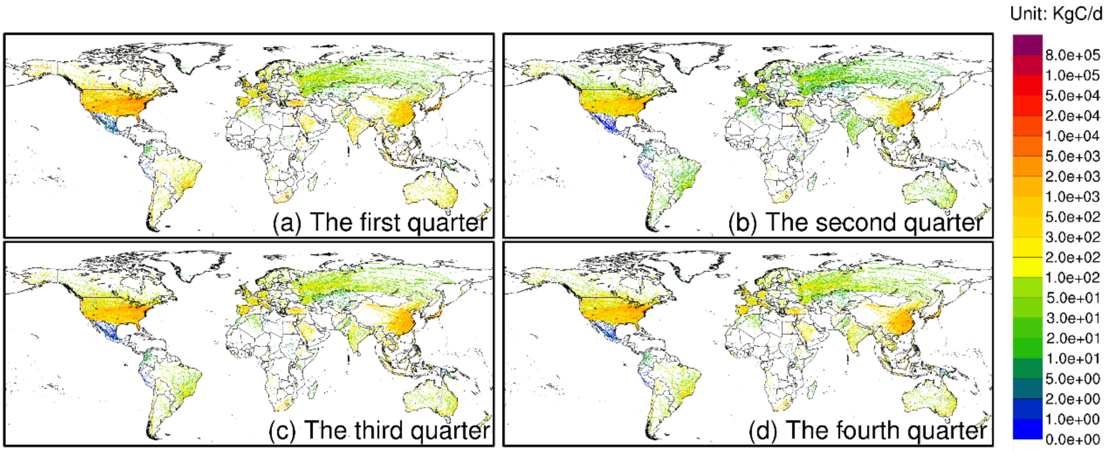

Figure S2(G). Per quarter daily mean emissions from **Domestic aviation** sector in 2020.

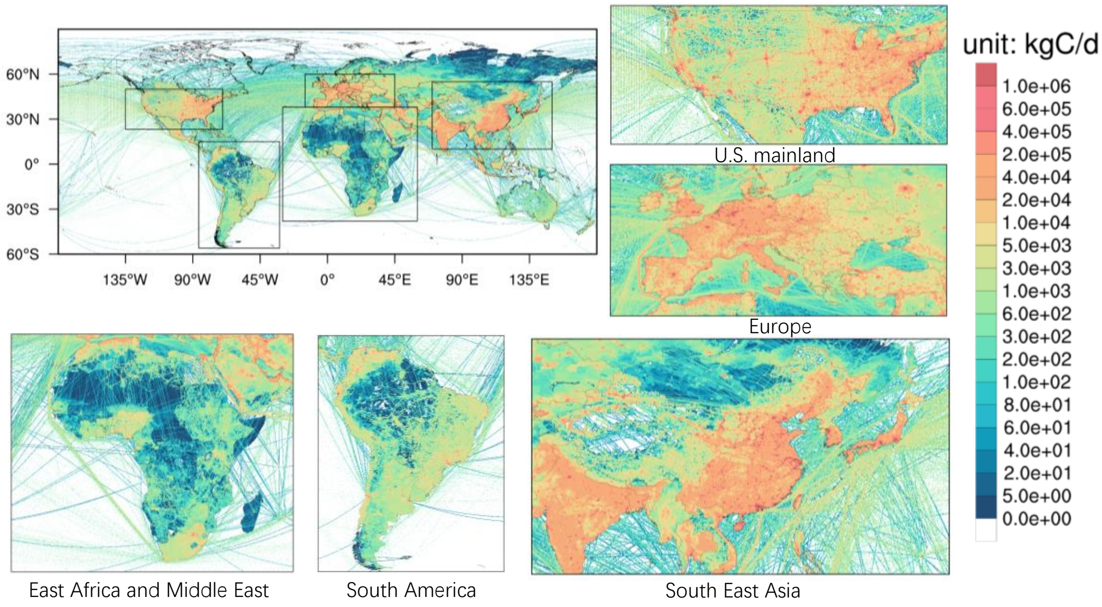

Figure S3. The range value of daily variations of total emissions in 2020.

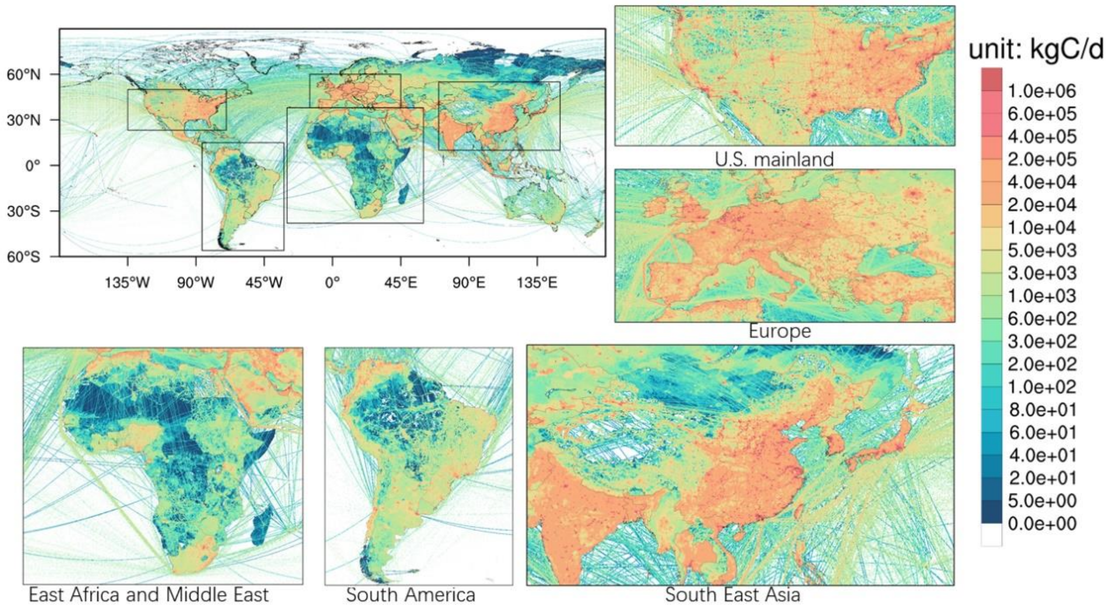

Figure S4. The range value of daily variations of total emissions in 2019.

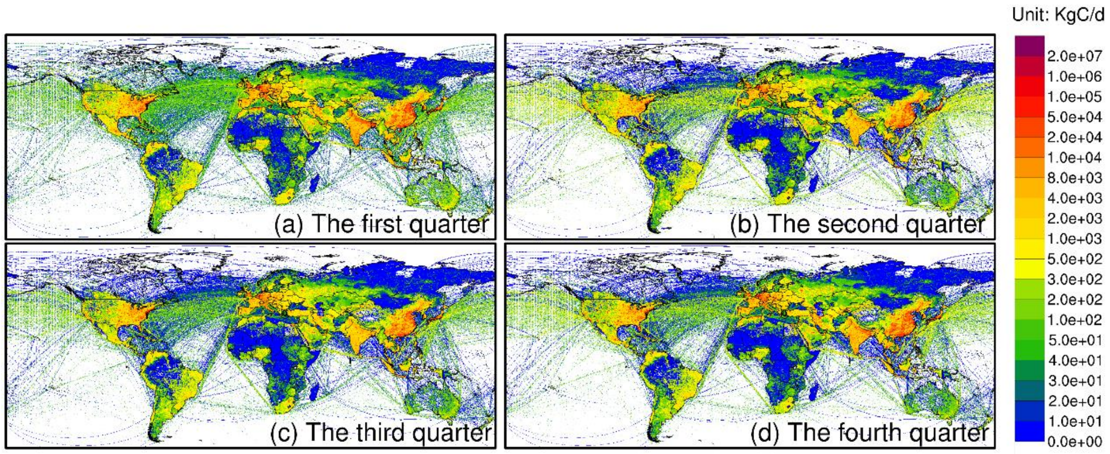

Figure S5. Maps of standard deviation of daily total emissions per quarter in 2020.

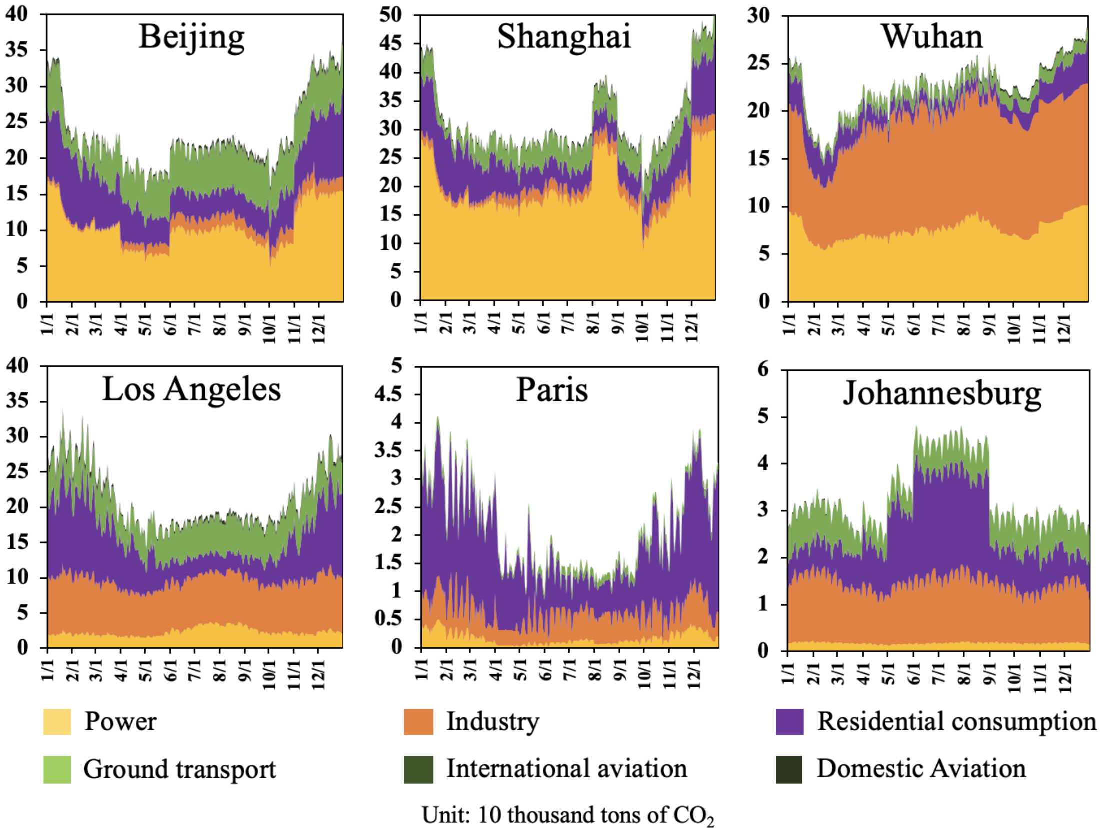

Figure S6. Graphs of daily sectoral emissions at the city-level in 2020.

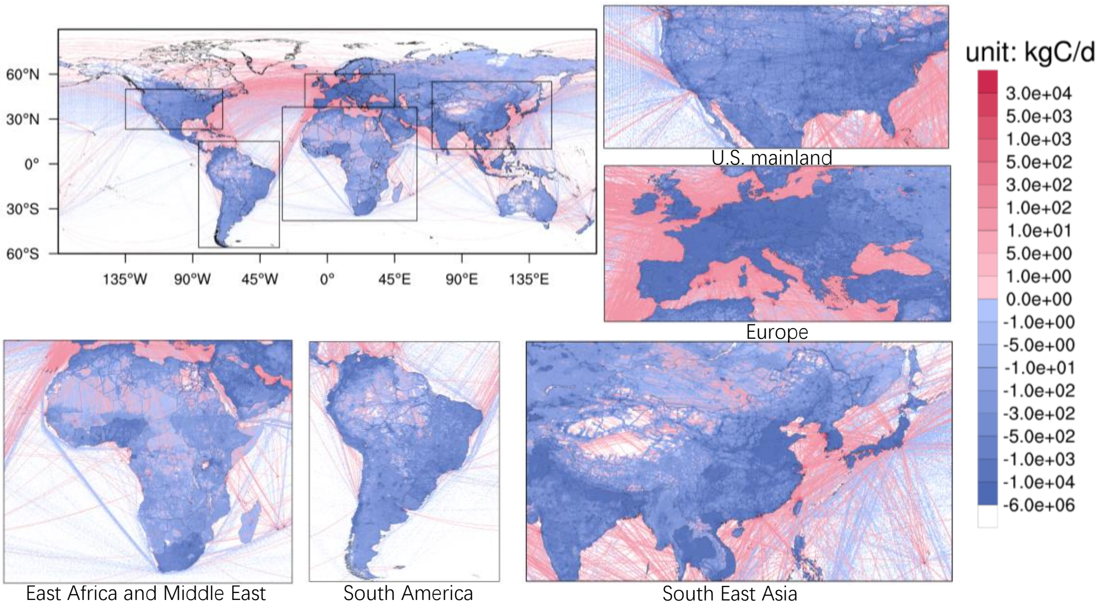

Figure S7. Map of weekend minus weekday emissions in 2019.

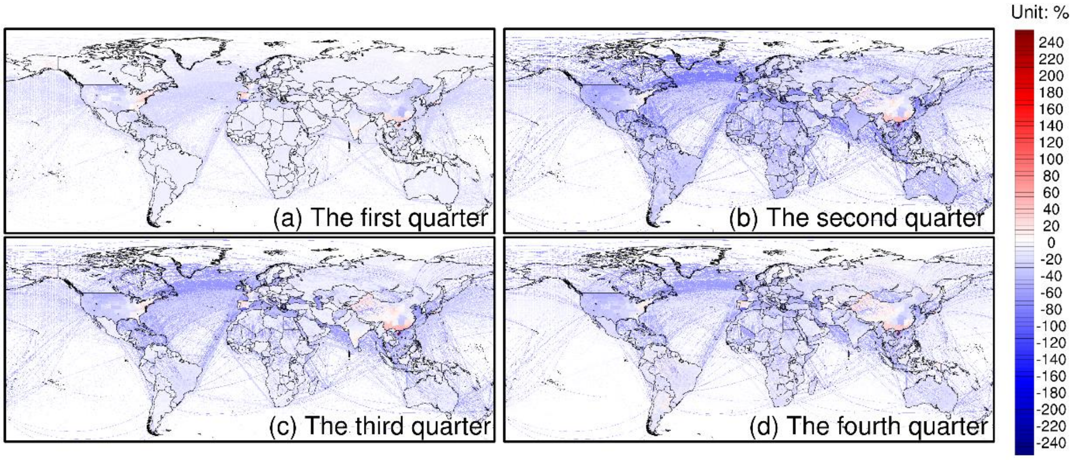

Figure S8. Maps of percent change in daily average CO_2 emissions between 2020 and 2019 per quarter. Note: the percent change is calculated by (2020-2019)/2019.

Figures S9 show the spatial distribution of the daily average GRACED emissions in 2020 by sectoral category. The spatial distribution characteristics of emissions from various sectors show a great difference, which is mainly explained by the emission sources.

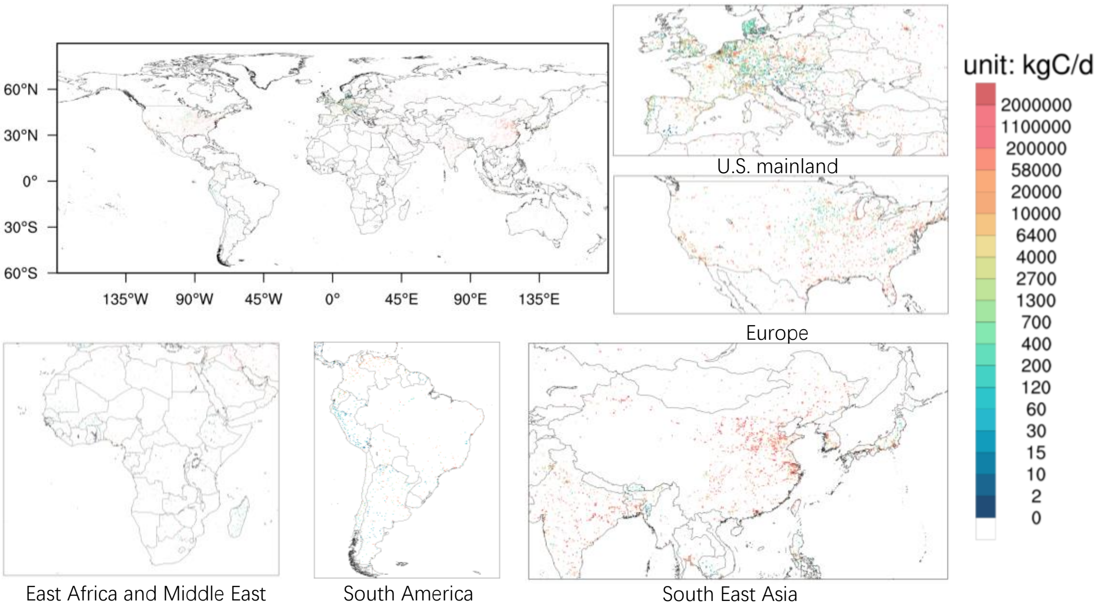

Figure S9(A). The sectoral CO_2 emissions distributions of GRACED in 2020 for **Power** sector. The values in the figures are given in the unit of Kg of carbon per day per cell.

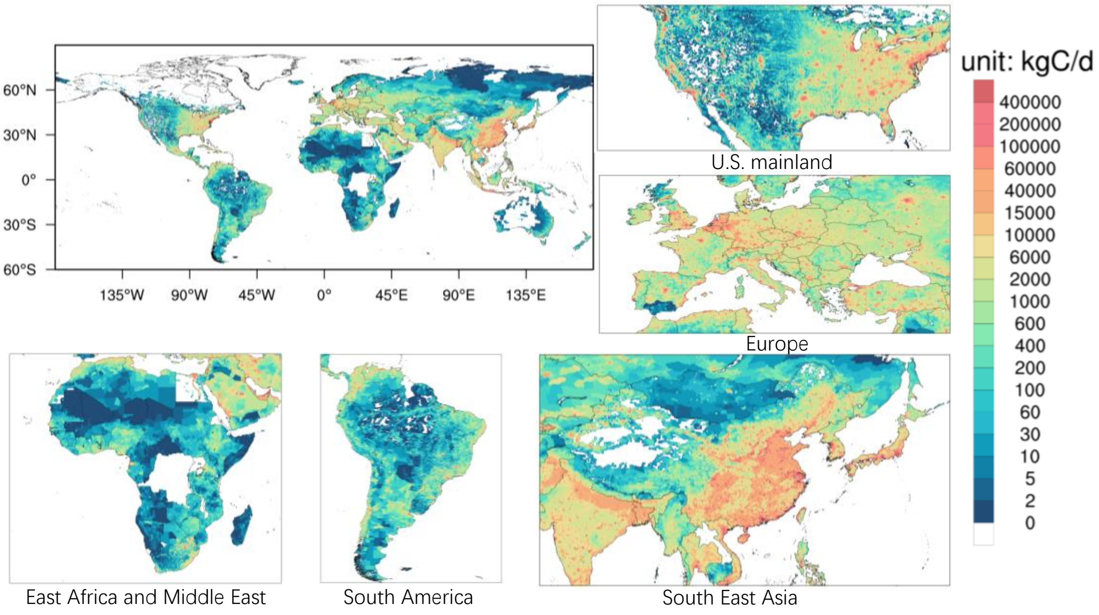

Figure S9(B). The sectoral CO_2 emissions distributions of GRACED in 2020 for **Industry** sector. The values in the figures are given in the unit of Kg of carbon per day per cell.

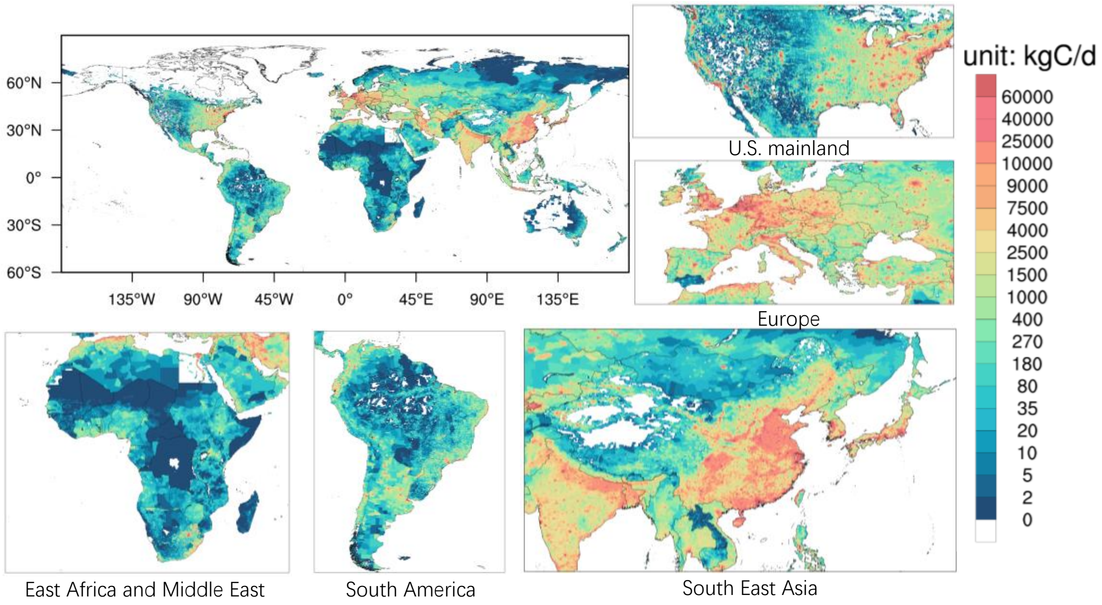

Figure S9(C). The sectoral CO_2 emissions distributions of GRACED in 2020 for **Residential Consumption** sector. The values in the figures are given in the unit of Kg of carbon per day per cell.

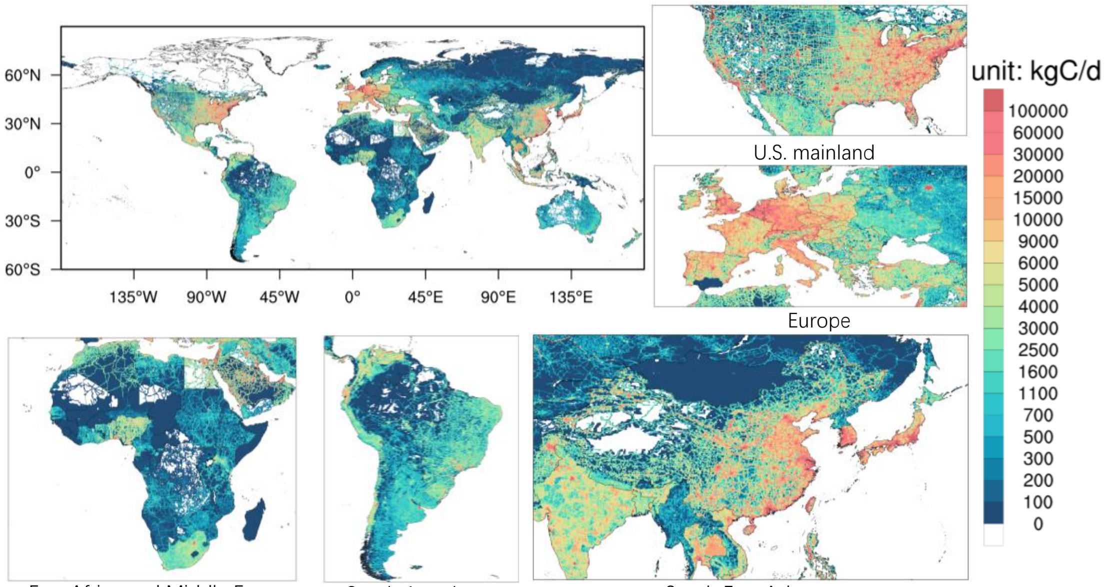

Figure S9(D). The sectoral CO_2 emissions distributions of GRACED in 2020 for **Ground Transport** sector. The values in the figures are given in the unit of Kg of carbon per day per cell.

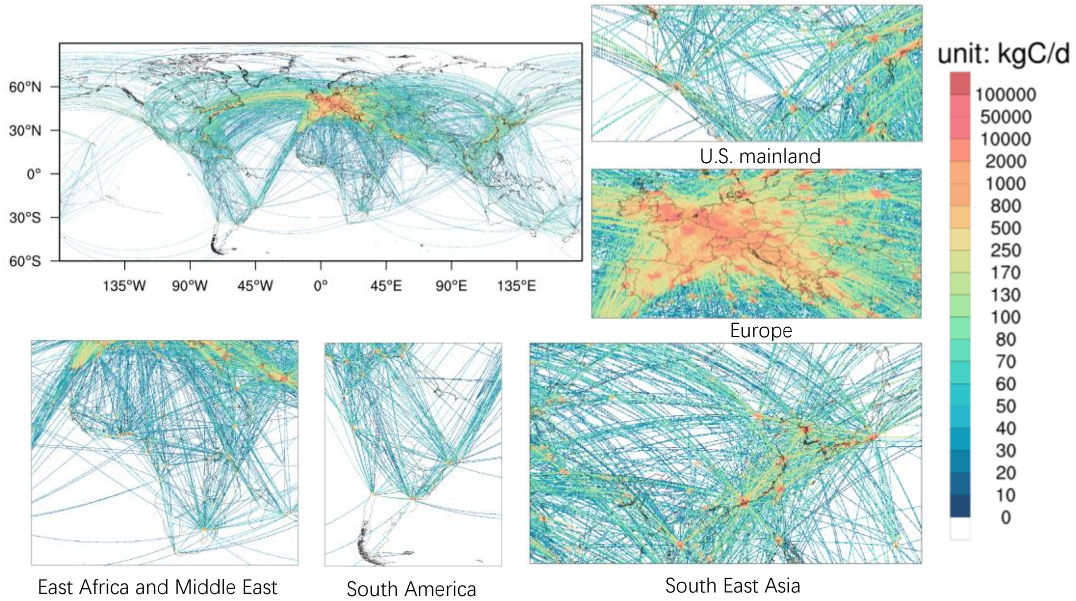

Figure S9(E). The sectoral CO_2 emissions distributions of GRACED in 2020 for **International Aviation** sector. The values in the figures are given in the unit of Kg of carbon per day per cell.

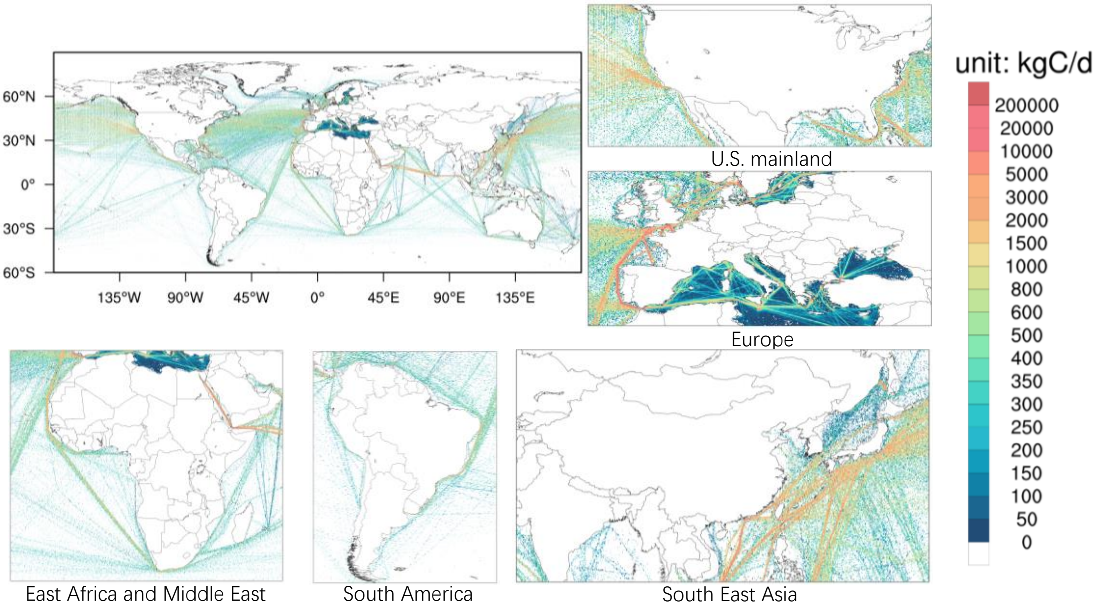

Figure S9(F). The sectoral CO_2 emissions distributions of GRACED in 2020 for **International Shipping** sector. The values in the figures are given in the unit of Kg of carbon per day per cell.

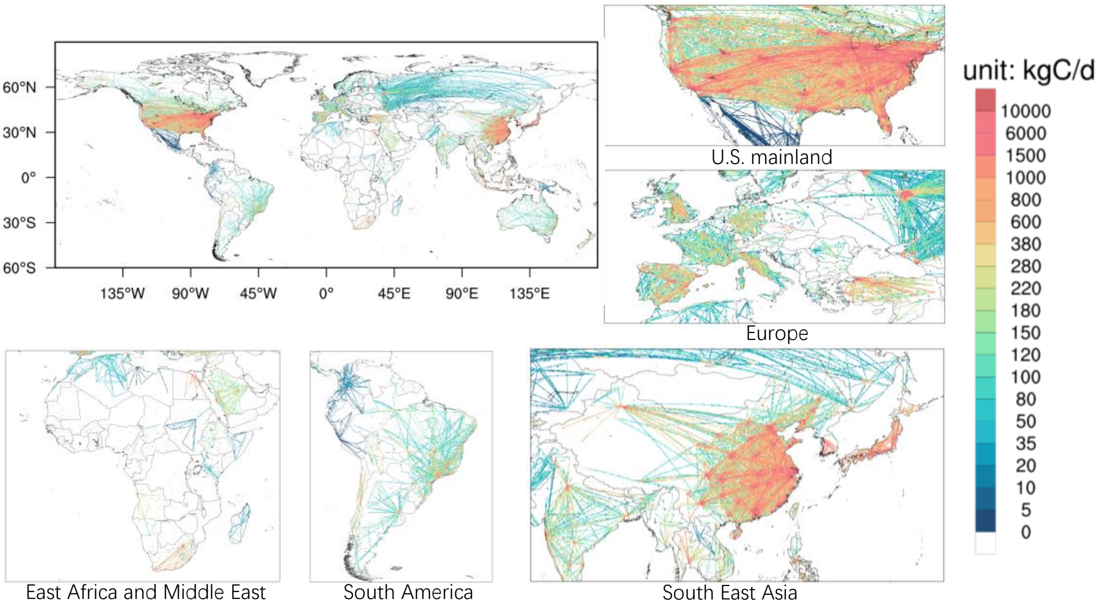

Figure S9(G). The sectoral CO_2 emissions distributions of GRACED in 2020 for **Domestic Aviation** sector. The values in the figures are given in the unit of Kg of carbon per day per cell.

Figures S10 show the map of weekend minus weekday emissions in 2020 by sectoral category. The spatial distribution characteristics of differences from various sectors show a great difference.

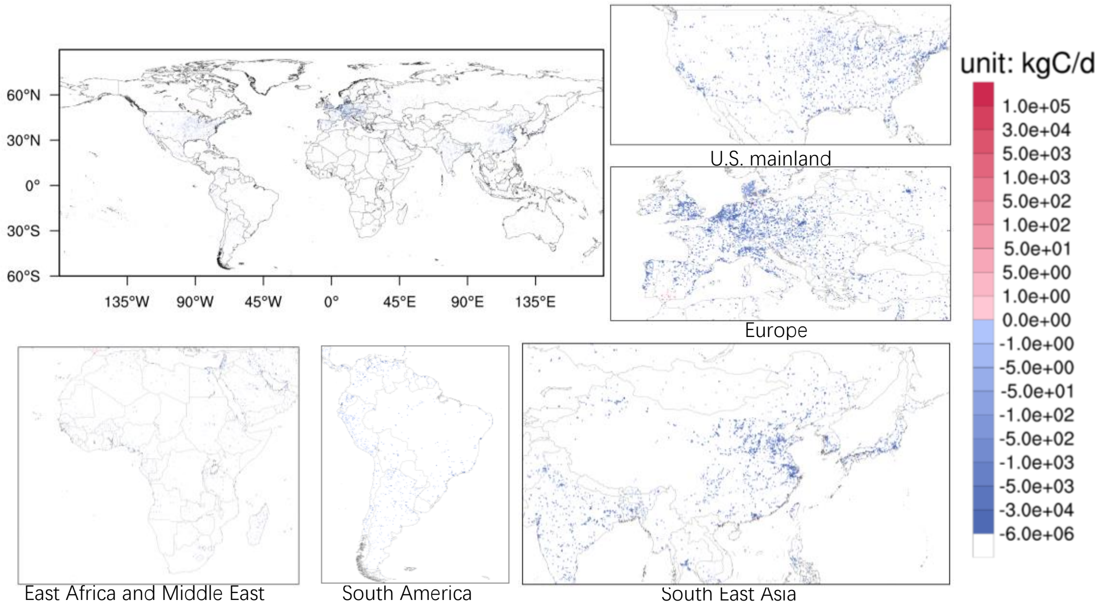

Figure S10(A). Map of weekend minus weekday emissions in 2020 for **Power** sector.

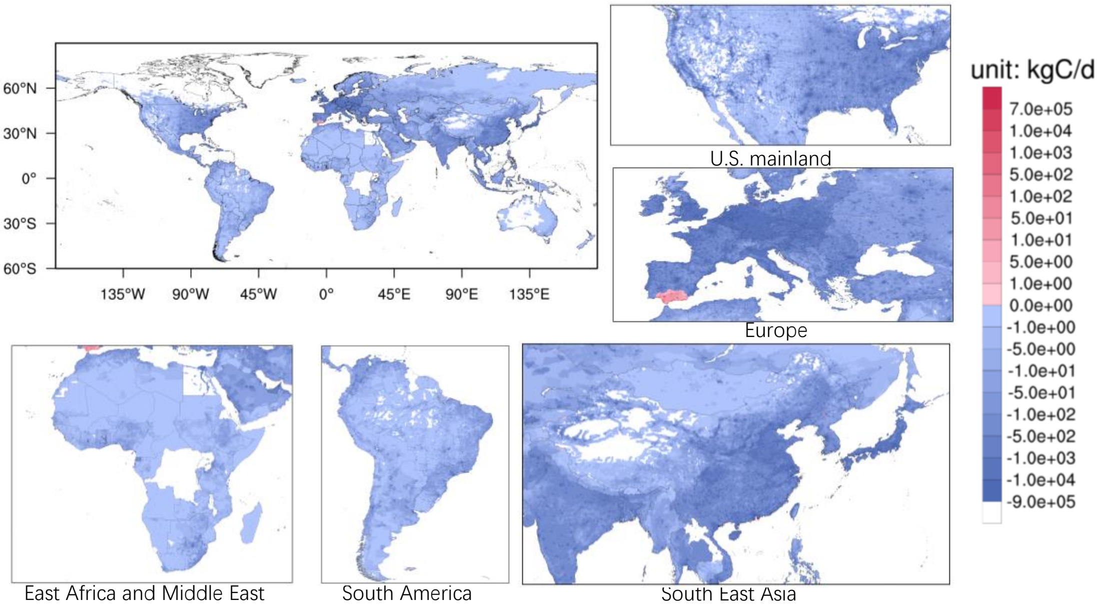

Figure S10(B). Map of weekend minus weekday emissions in 2020 for **Industry** sector.

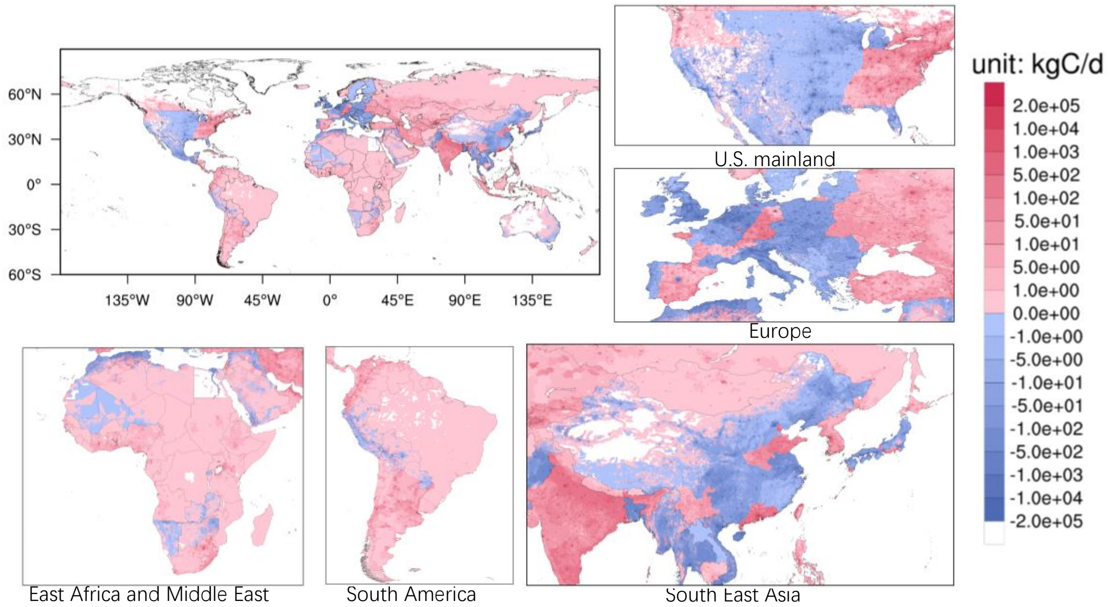

Figure S10(C). Map of weekend minus weekday emissions in 2020 for **Residential** sector.

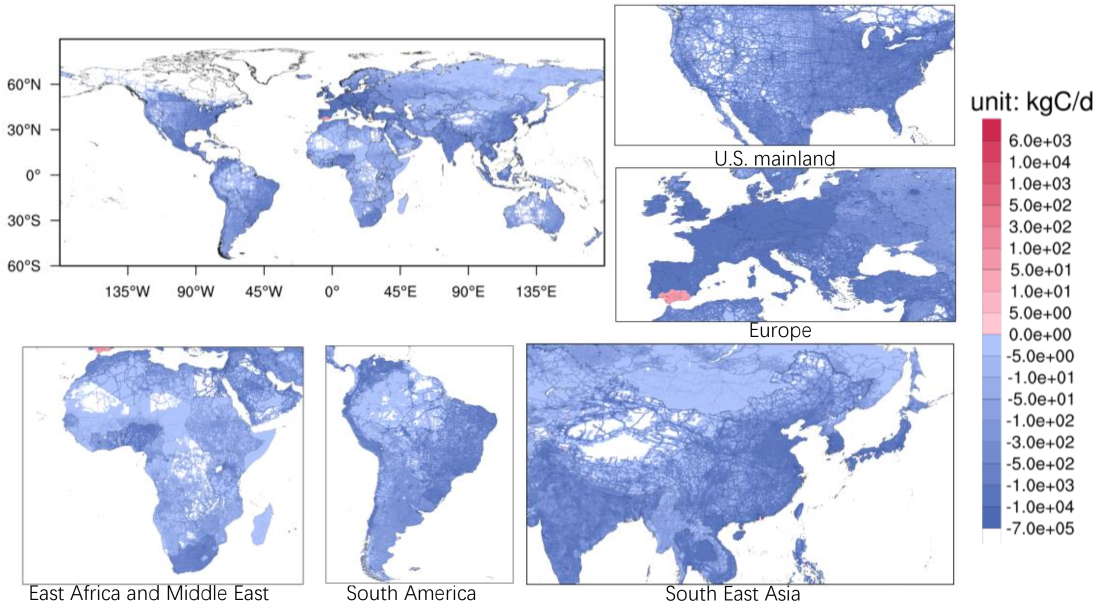

Figure S10(D). Map of weekend minus weekday emissions in 2020 for **Ground Transportation** sector.

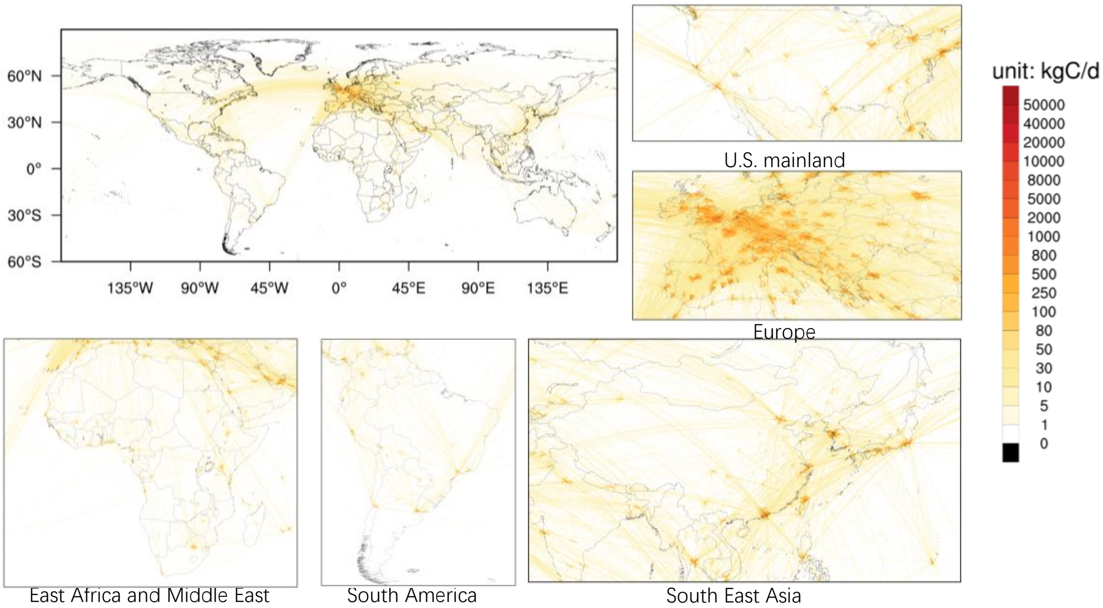

Figure S10(E). Map of weekend minus weekday emissions in 2020 for **International Aviation** sector.

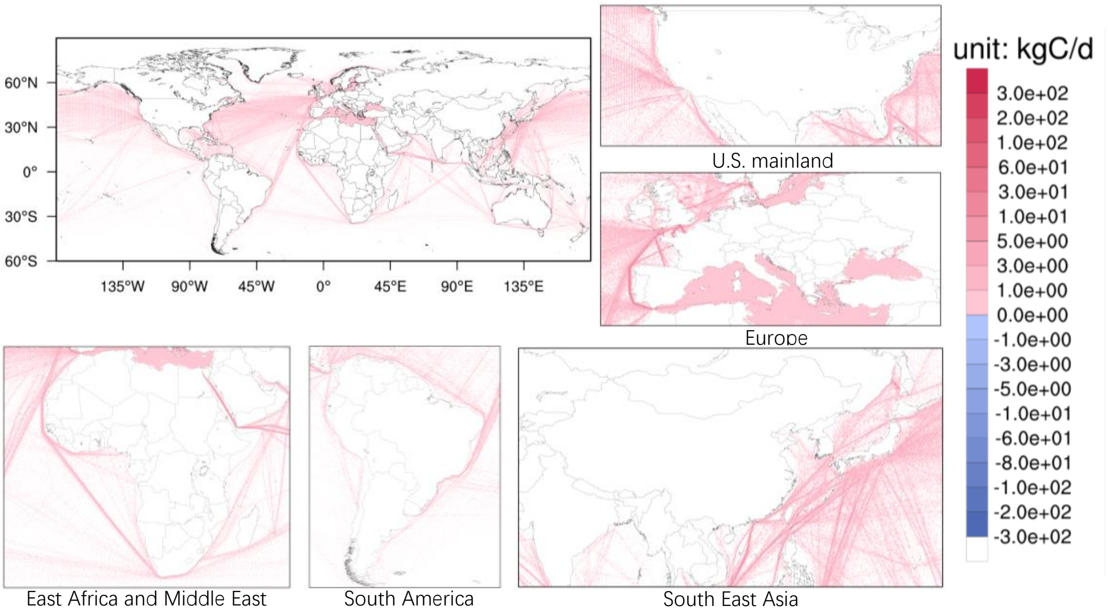

Figure S10(F). Map of weekend minus weekday emissions in 2020 for **International Shipping** sector.

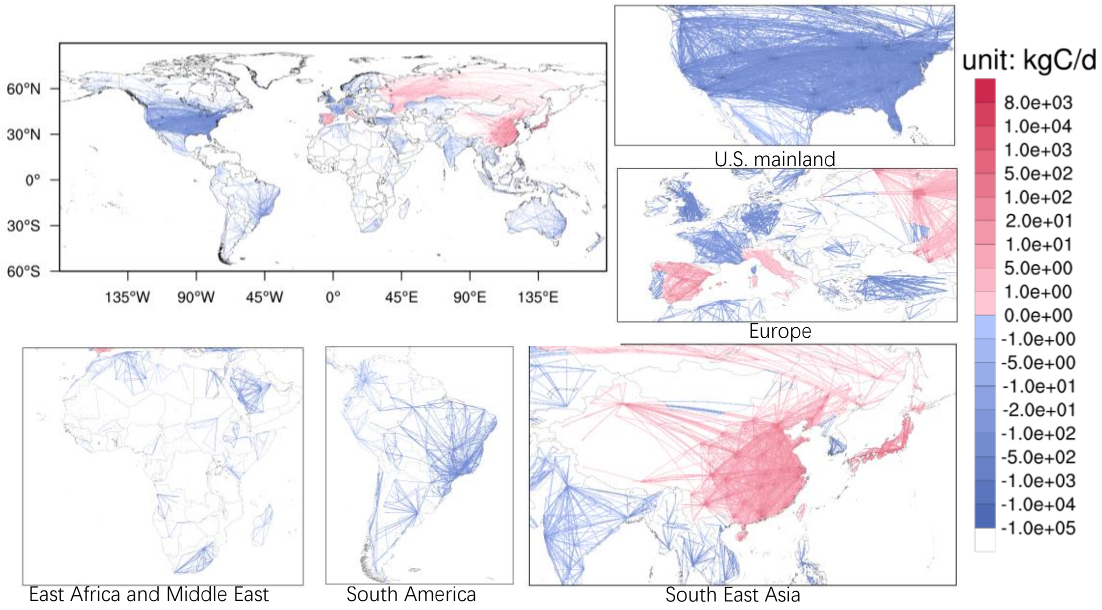

Figure S10(G). Map of weekend minus weekday emissions in 2020 for **Domestic Aviation** sector.

Supplemental Tables

Table S1. Correspondence between Carbon Monitor categories and GID/EDGAR categories.

No.	**Carbon Monitor categories**	**GID categories**	**EDGAR categories**
1	Power	power	
2	Industry	industry	
3	Residential consumption	resident	
4	Ground transport	transport	
5	International aviation		AIR Bunker oil for international transport
6	International shipping		SEA Bunker oil for international transport
7	Domestic aviation		AIR Bunker oil for domestic transport

Table S2. The relationship between the 7 super-sectors of GRACED and the IPCC sectors.

**IPCC**	**IPCC description**	**This study**
1A1a	Public electricity and heat production	Power

1A1bc	Other Energy Industries	Industry(incl. Cement Process)
1A2	Manufacturing Industries and Construction	Industry(incl. Cement Process)
2A1	Cement production	Industry(incl. Cement Process)
1A3a	Domestic aviation	Domestic aviation
1A3b	Road transportation no resuspension	Ground Transport
1A3c	Rail transportation	Ground Transport
1A3d	Inland navigation	Ground Transport
1A3e	Other transportation	Ground Transport
1A4	Residential and other sectors	Residential
1A5	Other Energy Industries	Residential
1C2	Memo: International navigation	International shipping
1C1	Memo: International aviation	International aviation

REFERENCES

1. Liu, Z., Ciais, P., Deng, Z., et al. (2020). Near-real-time monitoring of global CO2 emissions reveals the effects of the COVID-19 pandemic. Nature communications *11*, 1-12.
2. Liu, Z., Ciais, P., Deng, Z., et al. (2020). Carbon Monitor, a near-real-time daily dataset of global CO2 emission from fossil fuel and cement production. Nature Scientific Data *7*, 392, 10.1038/s41597-020-00708-7.
3. Liu, J., Tong, D., Zheng, Y., et al. (2021). Carbon and air pollutant emissions from China's cement industry 1990–2015: trends, evolution of technologies, and drivers. Atmospheric Chemistry and Physics *21*, 1627-1647.
4. Tong, D., Zhang, Q., Davis, S.J., et al. (2018). Targeted emission reductions from global super-polluting power plant units. Nature Sustainability *1*, 59-68.
5. Wang, X., Lei, Y., Yan, L., et al. (2019). A unit-based emission inventory of SO2, NOx and PM for the Chinese iron and steel industry from 2010 to 2015. Science of the total environment *676*, 18-30.
6. Zheng, B., Huo, H., Zhang, Q., et al. (2014). High-resolution mapping of vehicle emissions in China in 2008. Atmospheric Chemistry and Physics *14*, 9787-9805.
7. Janssens-Maenhout, G., Crippa, M., Guizzardi, D., et al. (2019). EDGAR v4. 3.2 Global Atlas of the three major greenhouse gas emissions for the period 1970–2012. Earth System Science Data *11*, 959-1002.

8. Crippa, M., Solazzo, E., Huang, G., et al. (2020). High resolution temporal profiles in the Emissions Database for Global Atmospheric Research (EDGAR). Nature Scientific Data ***7***, 1-17.
9. Crippa, M., Guizzardi, D., Muntean, M., et al. (2020). Fossil CO2 emissions of all world countries - 2020 Report 10.2760/143674.
10. Le Quéré, C., Jackson, R.B., Jones, M.W., et al. (2020). Temporary reduction in daily global CO2 emissions during the COVID-19 forced confinement. Nature Climate Change ***10***, 647-653.
11. Chevallier, F., Zheng, B., Broquet, G., et al. (2020). Local anomalies in the column-averaged dry air mole fractions of carbon dioxide across the globe during the first months of the coronavirus recession. Geophysical research letters ***47***, e2020GL090244.
